# Epidemiological Impact of GII.17 Human Noroviruses Associated With Attachment to Enterocytes

**DOI:** 10.3389/fmicb.2022.858245

**Published:** 2022-04-27

**Authors:** Marie Estienney, Georges Tarris, Nicole Abou-Hamad, Alain Rouleau, Wilfrid Boireau, Rémi Chassagnon, Siwar Ayouni, Philippe Daval-Frerot, Laurent Martin, Frédéric Bouyer, Jacques Le Pendu, Alexis de Rougemont, Gael Belliot

**Affiliations:** ^1^National Reference Centre for Gastroenteritis Viruses, Laboratory of Virology, University Hospital of Dijon, Dijon, France; ^2^UMR PAM A 02.102 Procédés Alimentaires et Microbiologiques, Université de Bourgogne, Franche-Comté/AgroSup Dijon, Dijon, France; ^3^Department of Pathology, University Hospital of Dijon, Dijon, France; ^4^Univ. Bourgogne Franche-Comté, INSERM, EFS BFC, UMR1098, Interactions Hôte-Greffon-Tumeur/Ingénierie Cellulaire et Génique, Dijon, France; ^5^Laboratoire Interdisciplinaire Carnot de Bourgogne, UMR CNRS 6303, Université Bourgogne Franche-Comté, Dijon, France; ^6^FEMTO-ST Institute, CNRS UMR-6174, Université de Bourgogne Franche-Comté, Besançon, France; ^7^Université de Nantes, Inserm, CRCINA, Nantes, France

**Keywords:** norovirus, evolution, HBGA, ligand affinity, duodenum

## Abstract

For the last 30 years, molecular surveys have shown that human norovirus (HuNoV), predominantly the GII.4 genotype, is one of the main causative agents of gastroenteritis. However, epidemiological surveys have revealed the worldwide emergence of GII.17 HuNoVs. Genetic analysis confirmed that GII.17 strains are distributed into three variants (i.e., Kawasaki 308, Kawasaki 323, and CS-E1). Here, virus-like particles (VLPs) were baculovirus-expressed from these variants to study putative interactions with HBGA. Qualitative analysis of the HBGA binding profile of each variant showed that the most recent and predominant GII.17 variant, Kawasaki 308, possesses a larger binding spectrum. The retrospective study of GII.17 strains documented before the emergence of the dominant Kawasaki 308 variant showed that the emergence of a new GII.17 variant could be related to an increased binding capacity toward HBGA. The use of duodenal histological sections confirmed that recognition of enterocytes involved HBGA for the three GII.17 variants. Finally, we observed that the relative affinity of recent GII.17 VLPs for HBGA remains lower than that of the GII.4-2012 variant. These observations suggest a model whereby a combination of virological factors, such as polymerase fidelity and increased affinity for HBGA, and immunological factors was responsible for the incomplete and non-persistent replacement of GII.4 by new GII.17 variants.

## Introduction

Each year, diarrheal diseases, such as viral gastroenteritis, affect millions of people of all ages around the world (Lozano et al., 2012). Human noroviruses (HuNoVs) have been recognized as one of the most predominant viral enteric pathogens (Ahmed et al., 2014; Wang et al., 2021). The growing routine use of real-time RT-PCR techniques for HuNoV detection and the establishment of efficient international networks for its surveillance has provided us with valuable information about its circulation. Complementary epidemiological studies have shown that young children and the elderly are most at risk of norovirus infection (Banyai et al., 2018). Human norovirus is considered highly infectious and is transmitted either person-to-person or through contaminated food and water. In the United States, the cost of infection associated to foodborne norovirus infections is estimated at roughly $2 billion per year (Scallan et al., 2011). Studies conducted in other parts of the world have reported similar figures, ranking it as the fifth foodborne hazard in terms of disability-adjusted life years (Belliot et al., 2014; Havelaar et al., 2015).

Norovirus is one of the 11 genera of the *Caliciviridae* and is currently divided into 10 genogroups (GI to GX; Chhabra et al., 2019; Vinje et al., 2019). Human noroviruses mostly belong to the GI, GII, and GIV genogroups and cause acute gastroenteritis. The GI, GII, and GIV genogroups are subdivided into 9, 27 and 3 genotypes, respectively (Chhabra et al., 2019). The increasing number of epidemiological studies and survey networks have clearly shown that GII.4 noroviruses were by far the most predominant throughout the world, with few exceptions (van Beek et al., 2018).

Histo-blood group antigens (HBGA), which include ABH and Lewis antigens, are involved in HuNoV attachment. For 80% of the European population, HBGA expression at the surface of enterocytes and in saliva is a common feature of the secretor phenotype. It is driven by the *FUT2* gene encoding the α1,2-fucosyltransferase, which is involved in the synthesis of the A, B, and H antigens, while the *FUT3* gene is responsible for the synthesis of Lewis b (Le^b^) and Lewis y (Le^y^) antigens. For 20% of the European population, the *FUT2* gene is inactivated by a point nonsense mutation, which abrogates the synthesis of the α1,2-fucosyltransferase. The homozygous recessive mutation is responsible for the absence of the ABH antigens and the non-secretor phenotype. Lewis a (Le^a^) and x (Le^x^) are still present in the saliva and at the surface of the enterocytes, provided that the *FUT3* gene is active. A volunteer study by Lindesmith et al. (2003) and later epidemiological surveys confirmed the strong correlation between the secretor phenotype and norovirus infections (Lindesmith et al., 2003; Kambhampati et al., 2016; Loureiro Tonini et al., 2020), whose binding profiles to HBGA are genotype- and variant-dependent (de Rougemont et al., 2011; Tenge et al., 2021). The strong affinity of noroviruses to HBGA and subsequent extended norovirus binding profiles could partly explain the prevalence of GII.4 over other genotypes (de Rougemont et al., 2011). Indeed, such as an increased affinity toward HBGA or higher mutational rates, could explain the high prevalence of GII.4 in epidemiological studies, such as an increased affinity toward HBGA or higher mutational rates, which then could explain the emergence of new recombinant strains that can evade the immune system (Donaldson et al., 2008; Bull et al., 2010; van Beek et al., 2018). The winter of 2014–2015 saw the emergence of the GII.17 genotype, first documented in the Republic of China (Fu et al., 2015; Lu et al., 2015). The GII.17 genotype also became predominant in Japan during the same period, but it remained sporadic outside of Asia (de Graaf et al., 2015; Matsushima et al., 2015). The oldest GII.17 variants were detected from archival stool samples dating back from 1978 (Rackoff et al., 2013). Since then, ORF2 genetic analyses showed that GII.17 noroviruses can be divided into three variants: the oldest variant which circulated mostly from 1978 to 2009 (variant CS-E1), the Kawasaki 323 variant circulating in 2013–2014, and the most recent Kawasaki 308 variant circulating since 2014 (Chan et al., 2015). The bulk of the variations between genogroups is carried by the P2 domain of VP1 (Zhang et al., 2015). A strong capacity for HBGA binding has only been demonstrated for the most recent GII.17 variant (i.e., Kawasaki 308 variant), which appears to be associated with new antigenic properties (Chan et al., 2015; Zhang et al., 2015; Jin et al., 2016). It has been hypothesized that the binding capacity recently acquired by the Kawasaki 308 variant is the result of the evolution of older strains with an optimization of the HBGA binding pocket (Singh et al., 2015; Jin et al., 2016; Qian et al., 2019). That being said, it is worth mentioning the existence of a 1976 GII.17 strain showing an identical binding site to the most recent GII.17 variant, suggesting that there were anterior preadapted variants, as it was recently proposed for GII.4 HuNoV (Mori et al., 2017; Ruis et al., 2020). The structural analyses of the GII.17 capsid showed that its binding pocket was similar to that of GII.4’s binding pocket (Koromyslova et al., 2017). Tyrosine residue at position 444 is involved in the α1,2 fucose interaction for both post-2000 GII.4 variants and GII.17 Kawasaki 308 variant, while the tyrosine residue is replaced by a valine residue in the 1978 GII.17 variant. The experimental replacement of the valine residue by a tyrosine residue at position 444 for the canonical 1978 variant induced a partial recovery of the binding capacity of the P particles used in the assay, suggesting that the V444Y mutation correlated with a gain in binding capability for GII.17 (Qian et al., 2019). GII.17 circulated for a 2-year period as a predominant genotype, providing us with new opportunities to analyze mechanisms by which the GII.17 genotype was increasingly detected, especially in Asia. In the literature, conventional binding assays mostly using P particles suggested that only recent Kawasaki 308 efficiently bound HBGAs. Here, we also studied GII.17 interactions with HBGA using histological analyses and baculovirus-expressed VLPs to determine the binding capability of the older variants, CS-E1 and Kawasaki 323. The ancillary objective was to determine why GII.17 disappeared again to the benefit of GII.4 by measuring relative affinities to HBGA. We finally discuss the short predominance of GII.17 in the population and its interactions with HBGA.

## Materials and Methods

### Biological Materials

One hundred and two saliva samples and swabs of buccal epithelial cells were collected from individuals from French (*N* = 64) and Tunisian cohorts (*N* = 38). The use of the saliva for genetic analysis was approved by the Nantes University Hospital Review Board for the French cohort (study no. BRD02/2-P). Informed consent was obtained from all the donors. For the Tunisian cohort, the study was approved by the Ethics Committee of the Fattouma Bourguiba Public Hospital in Monastir (Tunisia) (committee decision of the 9th of May 2013), and informed consent was obtained from the parents of the involved children. Tissue specimens from bowel resection were obtained from the Pathology Department collection of the University Hospital of Dijon, for which the approval (reference 18.11.29.52329) was granted by the French national ethics committee (CPP19002).

### Norovirus ORF2 Cloning

The E12905 isolate is a GII.17 HuNoV similar to the epidemic Kawasaki 308 variant (Chan et al., 2015). The GII.17 strain (E12905, variant Kawasaki 308, GenBank number KU587626) was first amplified using 5′-TCCGCCCTGCAGATGAAGA TGGCGTCGAATG-3′ sense primer (PstI site is underlined) and RT 5′-TGGGTCGCGGCCGCTTACTGAGCCCTCCTT CG-3′ antisense primer (NotI site is underlined). Following amplification and digestion, the PCR product was resolved by electrophoresis, gel-purified and cloned into pVL1392 plasmid vector before transfection. The ORF2 of the GII.4 2012 variant was also cloned into pVL1392 plasmid vector. The primers used for the amplification of the 2012 variants were described previously (de Rougemont et al., 2011). Details about the cloning strategies are available upon request.

### Gene Synthesis

Gene synthesis and cloning were provided by Life Technologies (Saint-Aubin, France) and Genecust (Ellange, Luxembourg). The ORF2 coding sequence of GII.17-JC129 strain (GenBank number KY406981, variant CS-E1) and GII.17-CUHK-NS-360 strain (GenBank number KP902565, variant Kawasaki 323) were synthesized and cloned into pUC with PstI (CUHK-NS-360 strain) and BamHI (JC129 strains) restriction sites directly located upstream of the first ORF2 start codon. All constructs were engineered with NotI restriction site at the 3′ end, which was directly located downstream from the ORF2 stop codon. Following digestion and gel purification, the fragments were subcloned into plasmid vector pVL1392 (CUHK-NS-360 strains) and pVL1393 (JC129 strains).

### Production of Recombinant Baculovirus and Purification of the VLPs

Sf9 cells were used to generate recombinant baculoviruses by transfecting 10^6^ Sf9 cells with 1 μg of pVL vector and 200 ng of linearized BacPAK™6 DNA baculovirus genome (Clontech/Takara) using OPTIMEM medium and lipofectamine (Life Technologies, France), following manufacturer’s recommendations. The transfection procedure was allowed to run for 4 h at room temperature prior to replacing the mixture with 2 ml/well of fresh Grace’s medium (Sigma) supplemented with 10% fetal calf serum (FCS) (Life Technologies). The transfected cells were then incubated for 6 days at 27°C. Recombinant baculoviruses were directly purified by plaque assay and each clone was reamplified at low multiplicity of infection (MOI) on 10^6^ Sf9 cells implanted in six well plates. The infected cells were incubated for 6 days. The cell lysate was then harvested, and 50 μl of it was used to select the best clone for VLP production, using a ready-to-use immunoassay from RD-Biopharm (Saint-Didier au Mont d’Or, France) as described previously (de Rougemont et al., 2011). The titer of the cell lysate was also determined by plaque assay. To produce a large stock of inoculum, recombinant baculoviruses were propagated at 0.1 MOI on Sf9 cells using 10% FCS-Grace’s medium. For the VLP production, Hi5 cells were maintained in serum-free Express−5 medium supplemented with glutamine (Life Technologies). The Hi5 cells were inoculated with recombinant baculovirus at 2.5 MOI for 2 h at 27°C. The inoculum was then replaced by fresh medium and incubated for 6 days at 27°C. The cell lysate was then collected for concentration and purification as described previously (Belliot et al., 2001). The purified VLPs were diluted at a final concentration of 1 mg/ml in TNC buffer (10 mM Tris, 140 mM NaCl, 10 mM CaCl_2_, pH 7.4) containing 20 μg/ml of leupeptin (Sigma) prior to flash freezing in liquid nitrogen.

### Observation of VLPs by Electron Microscopy

The structure of VLPs was observed by high-angle annular dark-field scanning transmission electron microscopy (HAADF-STEM) using a JEOL JEM-2100F microscope operating at 200 kV. One drop of VLP sample was deposited on a carbon film-coated copper 300-mesh grid. After 1 min, the excess was drained off using a filter paper. Then, the sample was negatively stained with 1% ammonium molybdate (w/v). After 1 min, excess stain was removed and the sample was dried in air before STEM characterization.

### Expression and Purification of 1,2-α-L-Fucosidase Active Domain

The plasmid encoding the 1,2-α-L-fucosidase active domain was a gift from Takane Katayama (University of Kyoto, Japan). The enzyme was bacterially expressed in BL21 (DE3) delta-lacZ *Escherichia coli* following induction with 0.2 mM isopropyl β-D − 1-thiogalactopyranoside diluted in LB broth for 2 days at room temperature (Katayama et al., 2004). Soluble protein was extracted following sonication in ice water. Cell disruption was finalized by passing the cell lysate through a 5/8 in. gauge needle. Disrupted cells were then centrifuged at 10,000 *g* for 30 min at 4°C. Cleared supernatant was collected and expressed protein was purified on a nickel nitrilotriacetic acid column following manufacturer’s recommendation (Qiagen, France). Recombinant protein was eluted with 500 mM imidazole (Sigma, France) and was dialyzed overnight against 100 mM Na_2_HPO_4_ pH 6.5 containing 10% glycerol. Purified recombinant protein was aliquoted at 4 mg/ml, flash-frozen, and stored at −40°C until further use.

### VLP Binding Assay

Purified GII.17 VLPs from JC129 (CS-E1 variant), CUHK-NS-360 (Kawasaki 323 variant), and E12905 (Kawasaki 308 variant) were used for determining the binding profile of the three variants by using HBGA-phenotyped saliva (binding profile). The saliva binding assays were performed as described previously, using VLPs diluted at 5 μg/ml (de Rougemont et al., 2011).

For the affinity binding assays using glycoconjugates, lacto-N-fucopentaose I (LNFP-I, blood group H), A trisaccharide, and B trisaccharide were purchased conjugated to bovine serum albumin (BSA) (all from Dextra, United Kingdoms). An average of 20 synthetic HBGA ligands were covalently linked on each BSA molecule. Conjugated carbohydrates were serially diluted 2-fold in pH 9.6 carbonate/bicarbonate buffer and left overnight at 37°C. The plates were then washed three times with PBS buffer and incubated with 500 ng/well of purified VLPs diluted with PBS and 4% skimmed milk (PBS-4% blotto) for 1 h at 37°C. Each experiment was performed in triplicate. Purified 2012 variant GII.4 VLPs (GenBank number KM406485) were used as a positive control. Cloning and VLP production methods of the 2012 variant is similar to previous work (de Rougemont et al., 2011).

For saliva and affinity assays, attached GII.17 VLPs were then detected with rabbit polyclonal serum (gracious gift from bioMérieux, Marcy l’Etoile, France) raised against E12905 GII.17 isolates (Kawasaki 308 variant). The polyclonal serum was diluted 10,000 fold in PBS buffer-4% blotto and incubated for 1 h at 37°C prior to incubation with a 2,000-fold dilution of peroxidase-conjugated anti-rabbit antibody (Sigma, France) in PBS-4% blotto. Primary and secondary antibodies were each incubated for 1 h at 37°C. The chromogenic reaction was allowed to develop for 10 min at room temperature in the dark. The plates were read at 450–620 nm and the background was arbitrarily fixed at 0.2 OD.

### Surface Plasmon Resonance Analysis

The binding of the purified VLPs to Lewis x (Le^x^), LNFP-I, A, and B trisaccharides conjugated to BSA (all from Dextra) was analyzed by surface plasmon resonances (SPR) at 25°C with a Biacore 3,000 instrument (GE Healthcare) on homemade chips. Functionalization of the chips was described previously (de Rougemont et al., 2011). Briefly, the chips were chemically functionalized with a self-assembled monolayer composed of a mixture of 11-mercapto-1-undecanol and 16-mercapto-1-hexadecanoic acid at 1 mM (90/10 by mole) (Sigma-Aldrich: Saint-Quentin Fallavier, France). The sensor chips were cleaned with absolute ethanol (VWR: Le Périgare, France) then treated overnight and rinsed with ultra-pure ethanol and water (Elga LabWater: Antony, France). The carboxyl groups were activated by two injections for 7 min at 10 μl/min of 100 mM N-hydroxysulfosuccinimide sodium salt and 400 nM N-(3-dimethylaminopropyl)-N-ethylcarbodiimide (Sigma-Aldrich: Saint-Quentin Fallavier, France). The three glycoconjugates were diluted in 10 mM sodium acetate buffer and covalently linked to three separate channels on the same chip allowing the simultaneous analysis of the same VLP preparation. On average, 230 fmole/mm^2^ were linked on the sensorchip. Finally, free NHS sites were deactivated by one injection for 14 min at 10 μl/min with a solution of 1 M ethanolamine HCl (Biacore, GE Healthcare). VLPs were diluted in running HBS buffer (0.01 M HEPES, 0.15 M NaCl, 3 mM EDTA, and 0.005% surfactant P20 at pH 7.4) provided by the company, and were injected for 2 min at a flow rate of 10 μl/min and a concentration of 2 ng/μl. The injection was stopped and the dissociation was observed in running buffer for 7 min. The same chip was used for the analysis of two VLPs (GII.4 variant 2012 and GII.17 Kawasaki 308 variant). The chip was recycled by the injection of 5 μl at 10 μl/min of 10 mM glycine solution at pH 2.5 prior to new analysis.

### Competition Binding Assay

The saliva samples were coated, 1,000-fold diluted, in pH 9.6 carbonate/bicarbonate buffer, overnight at 37°C. The coated saliva was then treated with either 100 mM pH 6.5 sodium phosphate (negative control) or 8 μg/well of 1,2-α-L-fucosidase diluted in the same buffer, overnight at 37°C. After this step and the following steps, the plates were washed three times with PBS-Tween20 at 0.05%. The plate was then incubated with 2 μg/well of UEA-I and/or 2 μg/well of *Helix pomatia* agglutinin (HPA) (All from Sigma, France), overnight at 37°C. Non-specific sites were then blocked with PBS-blotto 4% for 1.5 h at 37°C. The same PBS-blotto buffer was used for the dilution of the VLPs and the antibodies. Five hundred nanogram/well of GII.17 VLPs were incubated for 2 h at 37°C prior to the incubation of the primary antibody (in-house GII.17-specific rabbit serum at dilution 10,000) for 1 h at 37°C. Bound primary antibodies were detected with an anti-rabbit IgG peroxidase-conjugated mAb (Sigma, France) for 1 h at 37°C at the dilution 2,000. Peroxidase activity was detected with 3,3′,5,5′ tetramethyl benzidine (KPL/Eurobio, Courtaboeuf, France). The reaction was stopped after 10 min incubation at room temperature with 1 N HCL prior to reading absorbance at 450 nm.

### Immunohistological Analysis

Three formalin-fixed paraffin-embedded (FFPE) tissue samples were selected from the Department of Pathology of the University Hospital of Dijon (France). The two duodenal samples were derived from a blood group O and a blood group A secretor (Le^a^-Le^b^ + phenotype) individual who underwent duodenopancreatectomy. The distal colon sample derived from a blood group A secretor patient who underwent colectomy. The samples were taken during routine surgical procedures performed at the University Hospital of Dijon (France). The detection of A, H, and Le^b^ antigens and the experimental conditions for VLP binding on histological sections were described previously using VLPs diluted at 5 μg/ml (Marionneau et al., 2002; Tarris et al., 2019); (Tarris et al., 2021). Attached VLPs were detected with GII.17-specific polyclonal serum described above. The serum was diluted 10,000 fold in PBS and incubated for 1 h at RT.

For HBGA detection and VLP binding assays, the primary antibodies were detected with anti-mouse and anti-rabbit peroxidase-conjugated antibodies (Sigma, United States), which both diluted 2,000 fold in PBS and incubated for 45 min at room temperature. Peroxidase activity was detected with a Vectastain® kit using 3,3′-Diaminobenzidine for 1.5 min at room temperature (Vector Labs, United States) before washing and counterstaining with hematoxylin (Dako, Agilent Technologies, United States). All histological sections were then dried and mounted with a cover slide in a Tissue-Tek Film® automaton. Slides were digitized using a Nanozoomer 2.0 HT slide scanner, then read using the NDP.view2 software (Hamamatsu, Japan). Whole slide images (WSI) are available upon request.

### Competition Experiments on Healthy Duodenal Histological Sections

To determine which HBGA is involved into the recognition of GII.17 on intestinal tissues, competition experiments using VLPs, enzymes, and monoclonal antibodies were performed. For the blood group O duodenal sample, histological sections were incubated with 6 μg 1,2-α-L-fucosidase diluted in 125 μl of 10 mM sodium phosphate buffer at pH 6.5 for 6 h at 37°C. The reaction mix was then renewed by a new batch of enzyme and incubated overnight at 37°C. Fucosidase-treated sections were either directly used for VLP binding assays as described above or preincubated with 4 μg of Le^b^-specific mAb in PBS, overnight at 37°C prior to VLP binding assays. Similar experiments using Le^b^-specific mAbs were also conducted on sections not treated with fucosidase. To control fucosidase activity, in a preliminary experiment, enzymatically treated sections were rinsed and directly incubated with FITC-labeled UEA-I diluted with PBS and incubated 1 h at 37°C (Supplementary Figure 1).

For the competition experiments using duodenal sections from a group A individual, the sections were either incubated with 10 μg of *Helix pomatia* agglutinin (HPA) in a final volume of 400 μl of PBS overnight at 4°C or treated with 1,2-α-L-fucosidase, as described above. VLP binding assays were then carried out as described above. In the final set of experiments, duodenal sections were first enzymatically treated prior to incubation with HPA and the VLP binding assay.

### Genetic Analysis

Four hundred seventy-five complete ORF2 amino acid sequences were retrieved from GenBank, of which 93 are unique sequences, and were used for genetic analysis. Alignment and sequence analysis were performed using the MEGA suite v.10 (Kumar et al., 2018). The sequences were first aligned using the MUSCLE algorithm, then manually checked for deletions. Homology between strains was estimated, based upon the number of amino acid changes, using MEGA without taking into account the deletions. Phylogenetic trees were generated from 1,000 replicates using the neighbor-joining and the maximum parsimony methods.

## Results

### Genetic Analysis

Epidemiological studies have documented the worldwide emergence of GII.17 norovirus strains, which became somewhat predominant in Asia during the years 2014–2015. When the GII.17 norovirus emerged, one could hypothesize that the GII.17 strains would definitively replace the GII.4 strains for the coming years (de Graaf et al., 2015). Our main objective was to characterize GII.17-HBGA interactions in order to assess whether the GII.17 genotype shares the features that were observed for GII.4.

**Figure 1:**
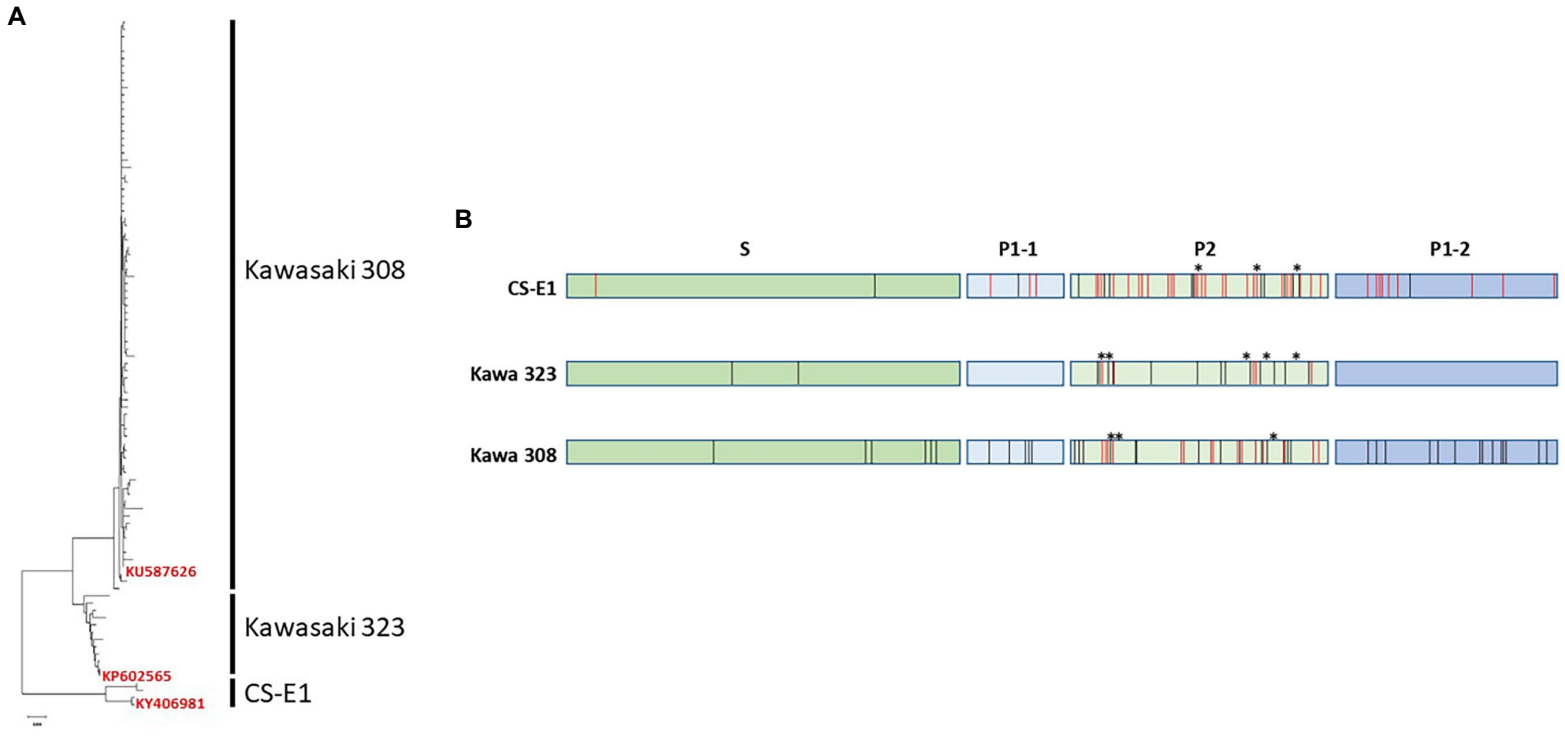
Genetic analysis of GII.17 human norovirus (HuNoV). **(A)** Neighbor-joining dendrogram of the 93 unique amino acid sequences corresponding to GII.17 ORF2. Variants are indicated on the right side of the dendrograms. For each variant, one isolate was selected for VLP production. The GenBank registration number of the isolate is indicated on the tree. **(B)** Summary of the alignment of the 93 amino acid sequences of GII.17. The sequences were divided into the three GII.17 variants, CS-E1, Kawasaki 323, and Kawasaki 308. S, P1, and P2 domains are indicated above the graph. Variations within each variant and between variant are indicated by black and red bars, respectively. Deleted amino acid residues are indicated by asterisks.

The genetic analysis of the 93 unique sequences of GII.17 was performed to determine whether the GII.17 genotype could be divided into variants, similar to GII.4 (Green, 2007). Neighbor-joining- and maximum parsimony-based dendrograms show similar topology where three distinct variants were observed: Kawasaki 308, Kawasaki 323, and CS-E1 variants (Figure 1A), as described previously (Chan et al., 2015). The CS-E1 variant could be divided into two groups, 1978 and 2002, as proposed previously (Qian et al., 2019). However, the percentage of homology was too high to truly differentiate two variants within CS-E1. Additionally, we constructed a minimum spanning tree including all the complete GII.17 capsid sequences available on GenBank by the end of year 2018 (*N* = 475). The topology of the minimum spanning tree clearly showed three distinct variants within the GII.17 genotype: Kawasaki 308, Kawasaki 323, and CS-E1 variants (data not shown).

Kawasaki 323 variant was circulating before it was replaced by the Kawasaki 308 variant (Matsushima et al., 2015). The CS-E1 variant is the oldest to be described, with only few sequences registered in GenBank (Zheng et al., 2006). The amino acid homology within each variant ranges between 96.1 and 99.8%. The bulk of the variations were located within the P2 domain. Unlike the GII.4 variants where one amino acid insertion was present in the P2 domain of the post-2000 variants, several deletions/insertions are present in the P2 domain within the three GII.17 variants (Figure 1B). For the three variants, no amino acid deletion or insertion was observed in sequences belonging to the same variant. Deletion or insertion was only observed within the P2 domain during paired comparison of the three variants (CS-E1 versus Kawasaki 323 or Kawasaki 308 and Kawasaki 323 versus Kawasaki 308; Figure 1B). Kawasaki 308 and 323 variants were quite similar, with 93.8%–96.2% homology. The percentage identity was markedly lower with CS-E1 variant with homology ranging between 86.3% and 89.9%. Again, the changes between the three variants were mainly located within the variable P2 domain.

### VLP Production

**Figure 2:**
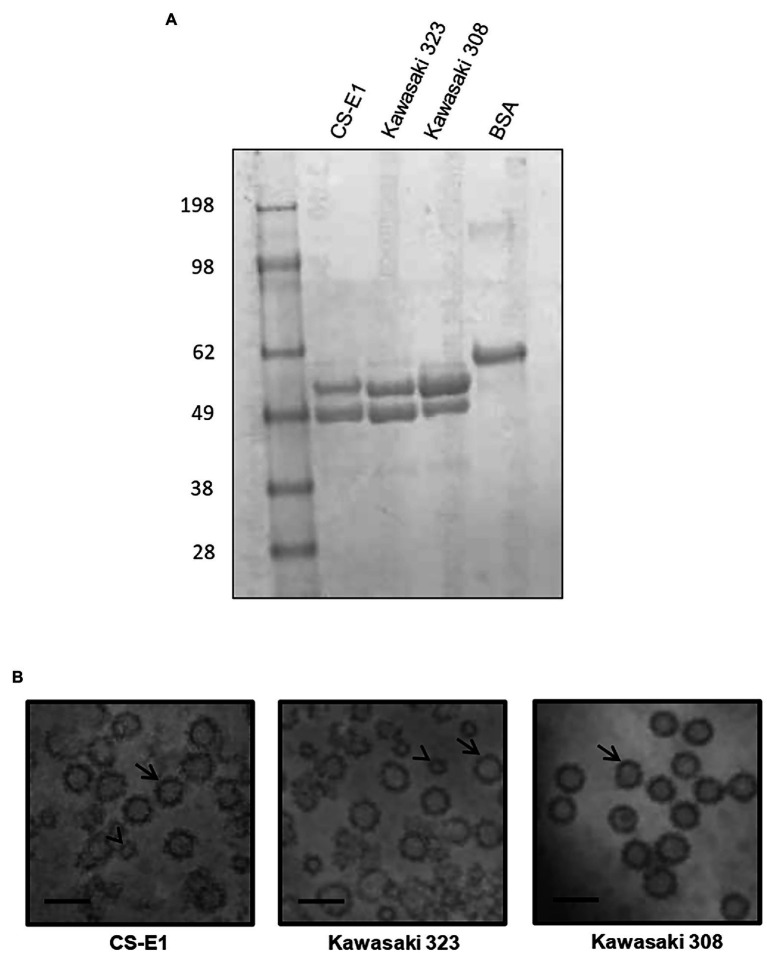
Virus-like particle (VLP) production. **(A)** Two micrograms of purified VLP were resolved on NuPAGE gel in denaturing conditions using MOPS buffer following manufacturer’s recommendations (Life technologies, France). The origin of each sample is indicated above the gel. Molecular weight markers are indicated on the left side of the gel. **(B)** Transmission electron microscopy images of GII.17 VLP preparations from scanning transmission electron microscopy. VP1_160_ (*T* = 3) and VP1_60-80_—like VLPs (*T* = 1) are indicated by arrow and arrowhead, respectively. Variants are indicated below each picture. The scale bar on each micrograph represents 50 nm.

To determine HBGA binding profiles, baculovirus-expressed VLPs were produced for each variant. At least a dozen of clones were plaque-purified following the transfection step for each variant. Small-scale VLP productions were then assayed by ELISA and partially purified proteins were resolved on NUPAGE gel (Figure 2A). A large production of VLPs for each variant was undertaken prior to their purification onto a cesium chloride gradient, as described previously (Belliot et al., 2001). A doublet corresponding to the complete and truncated forms of the VP1 protein was observed, as described previously for other genotypes (Belliot et al., 2001). The CS-E1 and Kawasaki 323 batches had a similar mix of complete VLPs and reduced size VLPs, which are equivalent to VP1_160_ (*T* = 3) and VP1_60-80_ (*T* = 1), respectively, as described previously (Shoemaker et al., 2010; Figure 2B). Kawasaki 308 VLPs were very homogeneous on electron microscopy and was only composed of complete VLPs (Figure 2B). For each preparation, arch-like organization of VP1 dimers was clearly observed by HAADF-STEM.

### GII.17 Binding Assay

**Figure 3:**
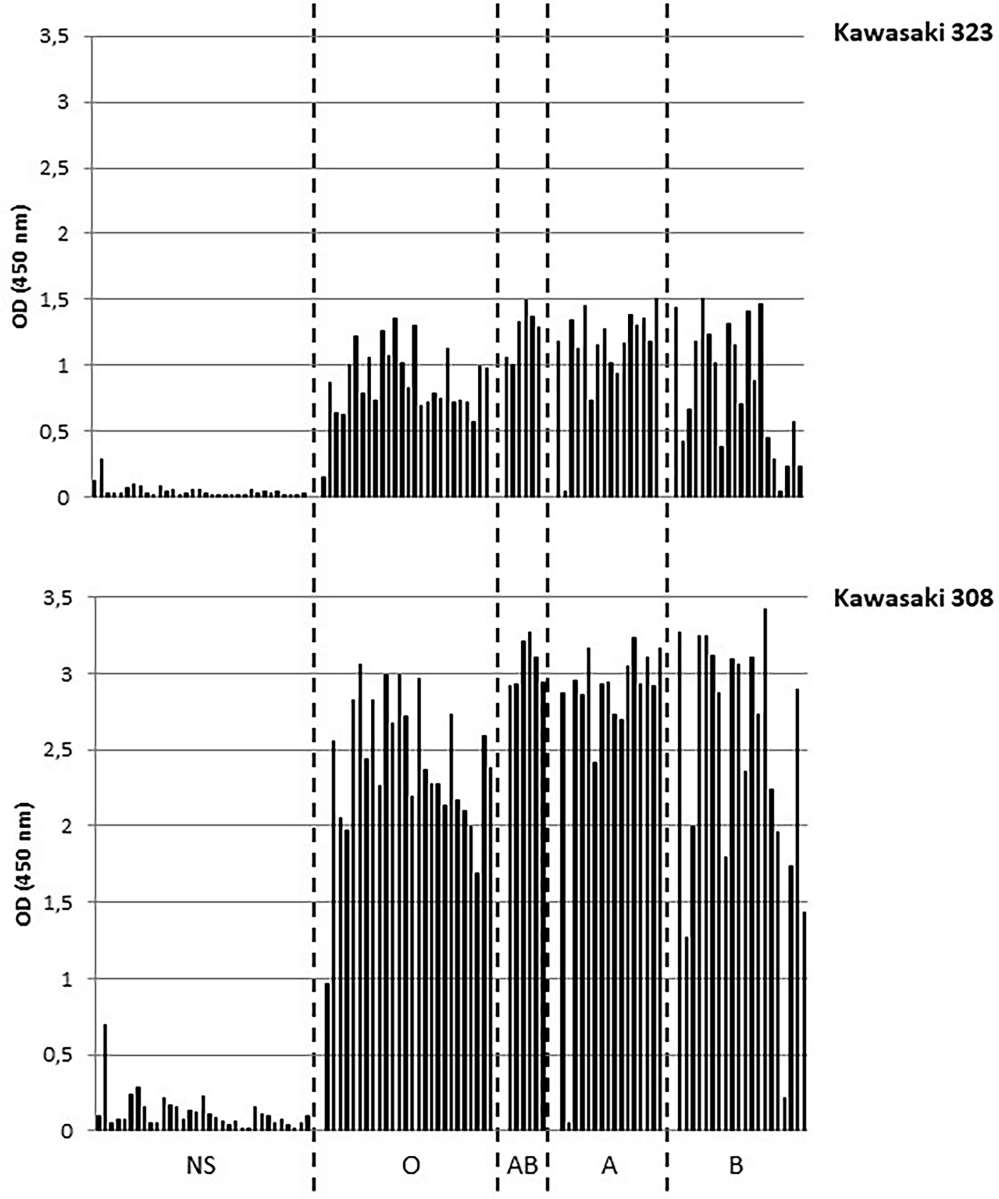
Saliva binding assays. The binding of purified VLP was measured by ELISA. The experiments were performed in duplicate for each saliva sample and the mean values are shown on the graph. OD values at 450 nm wavelength are indicated on the *y*-axis for this graph and the following. The non-secretor saliva (NS) and the ABH(O) blood group for saliva from secretor individuals are indicated below the graph and are separated by dashed lines. The name of the variant is indicated on the right side of the graph.

We first determined the saliva binding profile of the GII.17 variants using a panel of phenotyped saliva from secretor and non-secretor individuals (de Rougemont et al., 2011; Ayouni et al., 2015). Interestingly, the binding of Kawasaki 308 variant to ABH(O) antigens was previously demonstrated during a saliva binding assay using native particles from clarified stool (Chan et al., 2015). For our experiments, we used CsCl-purified VLPs. No binding was observed for the CS-E1 variant using 1,000-fold diluted saliva samples, even though the VLPs seemed structurally sound by electron microscopy. For Kawasaki 308 and 323 variants, we observed strong binding for ABO saliva, which was independent from the Lewis status, while no significant binding was observed with saliva from non-secretor individuals (Figure 3). Additionally, we observed that OD values obtained with Kawasaki 308 variant were higher but not statistically significant to those observed with Kawasaki 323 variant. This first set of data suggested that the relative affinity toward HBGA chronologically increased with the emergence of recent GII.17 variants based upon saliva binding profile, concordant with previous observations made using P particles (Jin et al., 2016).

### GII.4–GII.17 Relative Binding Comparison

**Figure 4:**
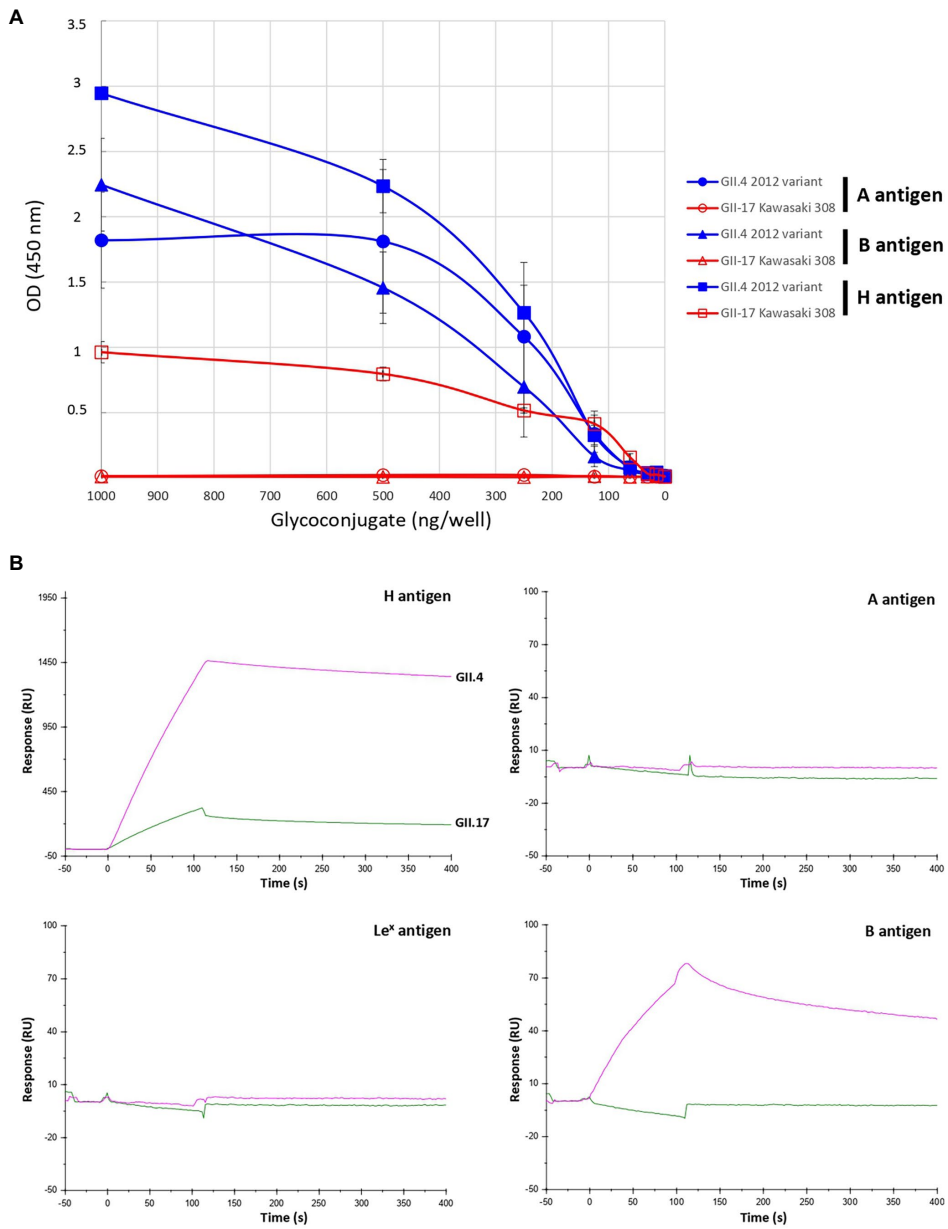
Relative binding of GII.17 Kawasaki 308 variant and GII.4 2012 variant to BSA-conjugated A, B, and H type 1 antigens by ELISA **(A)** and SPR **(B)**. For the ELISA binding assay, the conjugated histo-blood group antigen (HBGA) were diluted 2-fold in pH 9.6 carbonate–bicarbonate buffer from 1,000 to 15 ng per well. BSA-conjugated A, B, and H (LNFP-I) antigens are indicated by circle, triangle and square, respectively. The quantity of conjugated carbohydrates is indicated below the graph. HBGA and VLP genotypes for each experiment are indicated on the right side of the graph. Values are given in optical density at 450 nm wavelength (ordinate). Each binding experiment was performed in triplicate and the mean results are given with standard deviations (vertical bars). For the SPR binding assay, the same VLP and neoglycoconjugate preparations were used as for the ELISA. The response (ordinate) is given in resonance units (RU). The time (abscissa) is given in second (s). The first 120 s (*t* = 0 s through *t* = +120 s) correspond to the time of injection of the VLP. The sensorgram is color-coded for each variant, the name of which is indicated on the curves corresponding to the H antigen sensorgram. Pink and green curves correspond to the use of GII.4 (2012 variant) and GII.7 VLP (Kawasaki308 variant), respectively. The synthetic glycoconjugates used for the experiments are indicated on the upper right corner of each sensorgram.

Epidemiological surveys showed that GII.17 Kawasaki 308 variant circulated at the same time as the GII.4 2012 variant. In the next set of experiments, we compared the relative binding affinity of Kawasaki 308 to the GII.4 2012 variant for conjugated carbohydrates, as described previously (de Rougemont et al., 2011; Figure 4A). Variant GII.4 2012 VLPs efficiently attached A, B, and H synthetic antigens. The H antigen (LNFP-I) gave the highest values followed by A and B antigens. The three GII.17 variants were then assayed using the same A, B, and H synthetic carbohydrates. No binding was observed for Kawasaki 323 and CS-E1 variants for A, B, and H antigens (results not shown). For the Kawasaki 308 strain, specific binding was observed for the H antigen only, and the binding amplitude was markedly lower than that of the GII.4 2012 variant. Additionally, no binding was observed for A and B antigens (Figure 4A). The data were confirmed using SPR, in which the binding conditions are more stringent. Immobilized Le^x^ antigen was used as negative control and no binding was observed for GII.4 and GII.17 VLPs. First, we observed a very strong affinity of the GII.4 for the synthetic H antigen, whose amplitude was 3 times higher than that observed for GII.17. Here, the dissociation slope was higher for Kawasaki 308, suggesting a lesser affinity for the H antigen than that observed for the GII.4 2012 variant; VLPs were immobilized on the chip at 344 and 1,090 pg./mm^2^ for GII.17 and GII.4, respectively (Figure 4B). In addition, GII.4 2012 interacted strongly with antigen B but showed no binding with antigen A in these experimental conditions. We did not observe binding of the GII.4 2012 variant VLPs to A synthetic antigens unlike during the ELISA-based assay, likely due to the more stringent binding conditions in SPR experiments.

**Figure 5:**
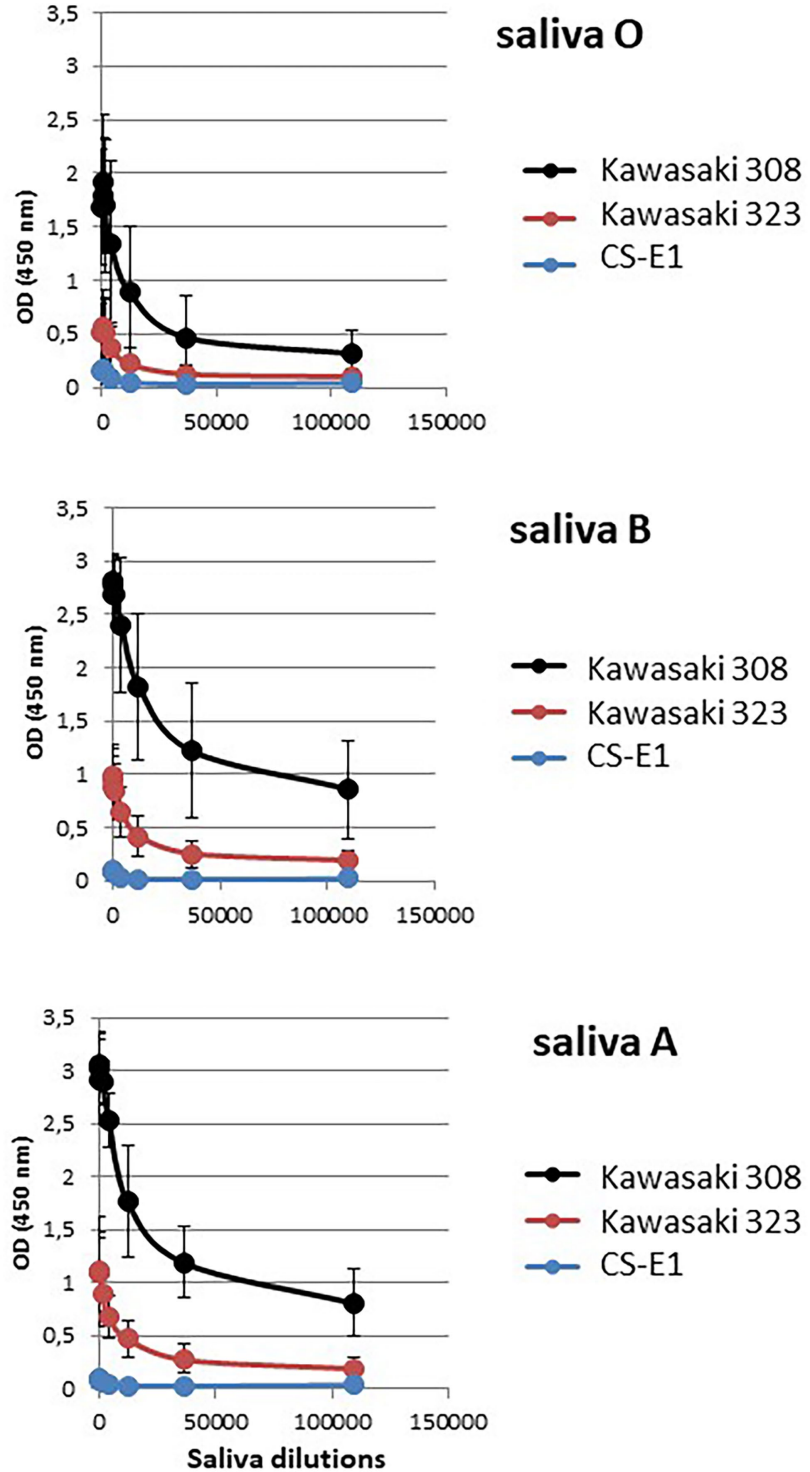
Relative affinity assay. A subset of six representative saliva samples for each ABO blood group was serially diluted by 3-fold and used to assess VLP affinity for each variant. Dilutions are indicated below the graph (abscissa). Each variant is color-coded, and the legend is indicated on the right side of each graph. Values are means of six individual experiments where standard deviations are shown by vertical bars. Blood groups are indicated on the left side of the graphs.

The absence or poor binding on synthetic carbohydrates for Kawasaki 308 and Kawasaki 323 variants was surprising when a marked attachment was observed using phenotyped saliva (Figure 3). This illustrates the fact that immobilized synthetic oligosaccharides do not fully mimic natural substances. Oligosaccharides are less dense than saliva mucins, so the backbone of the recognized motifs changes, as does their orientation. Therefore, to further characterize GII.17 binding toward HBGA, we performed a saliva binding assay using a subset of six phenotyped saliva samples for each blood group [i.e., A, B, and H(O) group], which were serially diluted 3-fold from 1/50 through 1/109350 for the ELISA binding assay (Figure 5). The analysis of each binding profile confirmed previous observations and clearly showed that VLP binding for each blood group was significantly higher for the Kawasaki 308 isolate than for the Kawasaki 323 and CS-E1 variants. Moreover, similar observations were made for the Kawasaki 323 isolate in comparison to the CS-E1 variant. Interestingly, CS-E1 VLP only produced faint binding with 50-fold diluted saliva. Overall, these experiments suggested that the binding capacity toward HBGA has increased with the recent evolution of GII.17 HuNoVs, with the highest binding activity observed for the Kawasaki 308 strain. Additionally, the data suggested that the binding capacity for the GII.17 Kawasaki 308 variant was lesser than that observed for the GII.4 2012 variant, which circulated at the same period. The next step was to ascertain recognition of HBGAs on native glycans by the GII.17 using Kawasaki 308 VLPs.

### GII.17 Binding Characterization

In the literature, structural studies showed that binding of the Kawasaki 323 and 308 variants involves the recognition of α1,2 fucose, which characterizes the H antigen (Singh et al., 2015; Koromyslova et al., 2017; Qian et al., 2019). In the first experiment, we used A, B, and O phenotyped saliva to determine the role of the α1,2 fucose moiety in the attachment of the Kawasaki 308 GII.17 VLPs. The fucosidase activity was checked by preincubating healthy duodenal tissue with the enzyme. The presence or absence of the α1,2 fucose moiety was ascertained using an H antigen-specific lectin (i.e., UEA-I lectin; Supplementary Figure 1). The UEA-I lectin specifically recognized the α1,2 fucose moiety characterizing the H antigen (Matsumoto and Osawa, 1969). The total absence of binding of the lectin following the enzyme treatment demonstrated that the fucosidase was indeed active.

**Figure 6:**
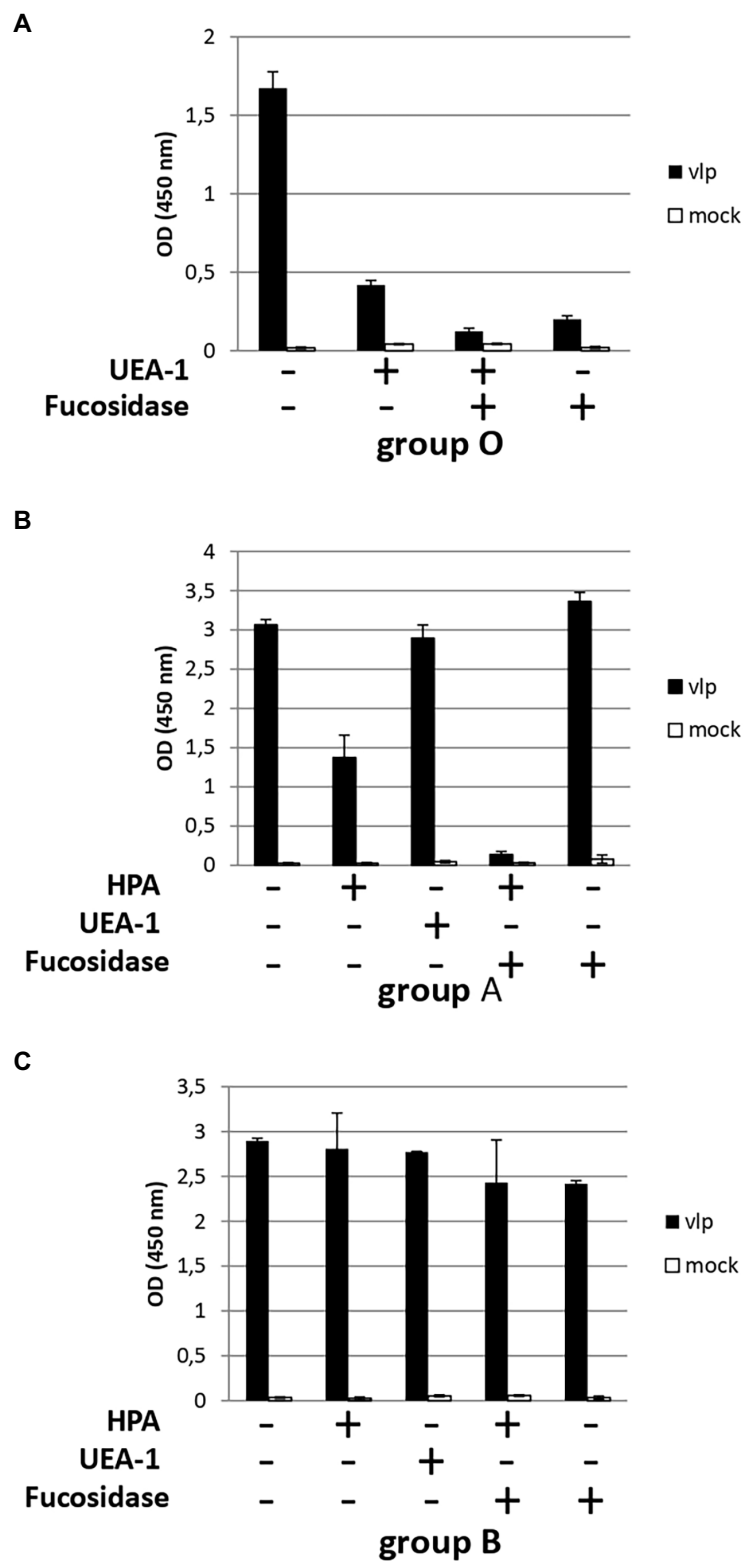
HBGA characterization involved in the recognition of GII.17. Diluted saliva samples from ABO patients were coated on ELISA plate prior to incubation with a combination of lectins (HPA and UEA-I) and 1,2-α-L-fucosidase. For each graph, the presence or absence of lectins and/or fucosidase is indicated below each graph by plus and minus signs, respectively. Blood group saliva is indicated below each graph. **(A)** For this experiment (with group O saliva) and the following experiments, diluted saliva was first incubated with 1,2-α-L-fucosidase and/or lectin prior to incubation of the coated saliva with VLPs (black bars) or PBS (mock, white bars). **(B)** Group A saliva. **(C)** Group B saliva.

In the first set of experiments, saliva samples from the ABO group were either directly used for salivary binding assays with Kawasaki 308 VLPs (positive control) or incubated with fucosidase and/or lectins (UEA-I and HPA) prior to VLP binding (Figure 6). HPA specifically recognized N-Acetylgalactosamine characterizing group A antigen (Prokop et al., 1968). Similar experiments were also conducted without VLPs and used as negative controls. The incubation of UEA-I lectin with O saliva reduced VLP binding by three-fold (Figure 6A). This observation was confirmed with the use of 1,2-α-L-fucosidase alone or combined with UEA-I lectin. In this case, VLP binding was almost entirely suppressed. Our data show that the α1,2 fucose moiety plays a pivotal role into the recognition of the GII.17 VLPs. In the next set of experiments, A and B saliva samples were subjected to a combination of fucosidase treatment and incubation with H- and A-specific lectins. For the group A saliva, the samples were either treated with fucosidase, lectins (UEA-I and HPA), or both (Figure 6B). The incubation of HPA reduced VLP binding by half while the α1,2 fucose moiety was still present. The fucosidase treatment alone or the preincubation of the saliva with UEA-I did not abolish VLP binding. Our observations were coherent with the literature which documents the poor efficacy of UEA-I and 1,2-α-L-fucosidase for the recognition of the α1,2 fucose on A and B antigens (Matsui et al., 2001; Katayama et al., 2004). However, the combination of 1,2-α-L-fucosidase and HPA totally abolished VLP binding on A saliva, suggesting that there is a trace of fucosidase activity. Our data again demonstrate that α1,2 fucose and N-acetyl-galactosamine moieties are required for the attachment of GII.17 as the structural analyses predicted using P particles (Singh et al., 2015; Koromyslova et al., 2017; Qian et al., 2019). Finally, no inhibition was observed when B saliva was incubated with HPA confirming that HPA specifically bound A saliva and that VLP inhibition was not merely the effect of the addition of a protein (Figure 6C).

### Histological Analysis of VLP Binding to Duodenal and Colonic Tissues

**Figure 7:**
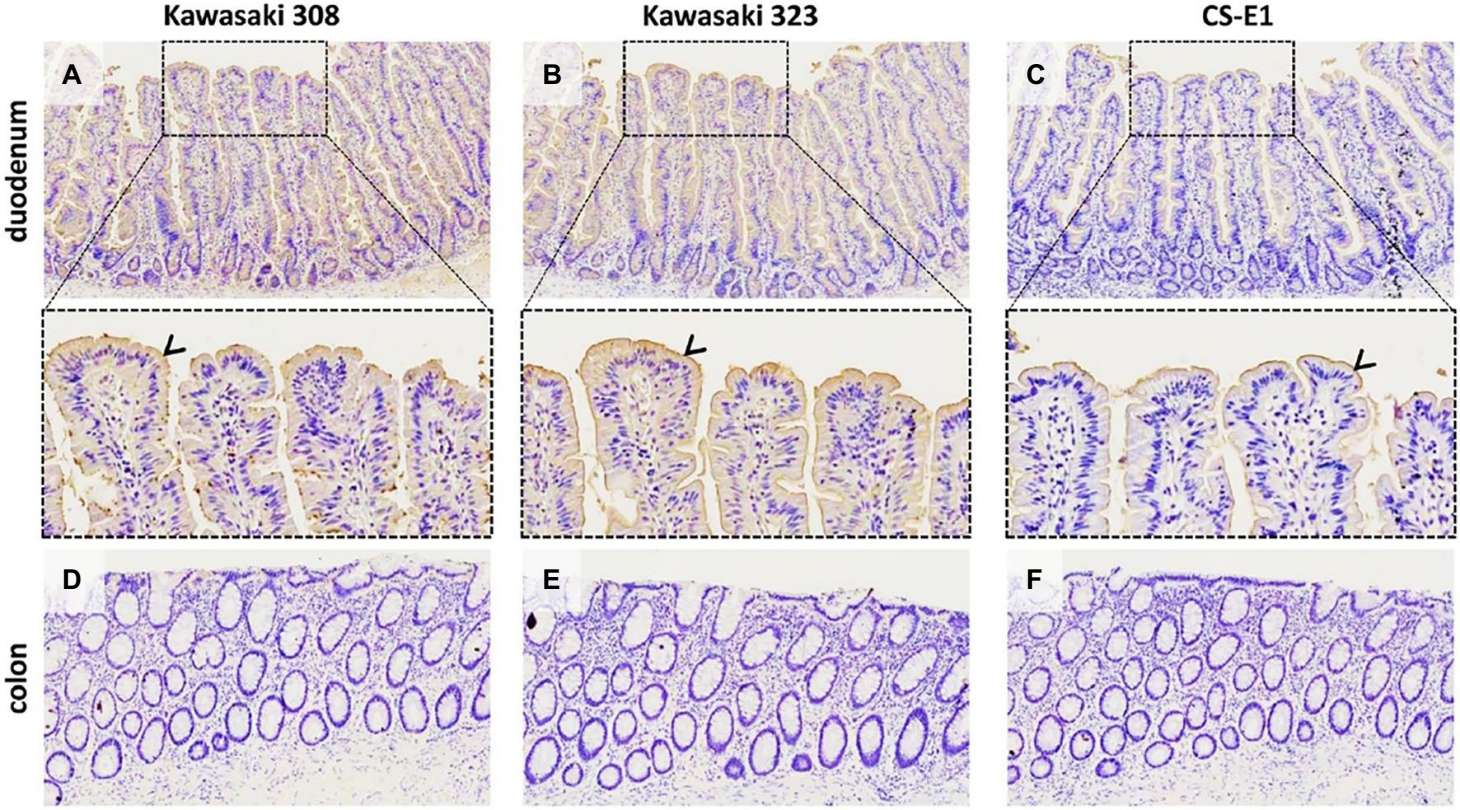
GII.17 attachment on duodenal and colonic histological sections from a AB blood group patient (magnification x100 for the first and last line). Duodenum (panels **A–C**) and distal colon (panels **D–F**) are indicated on the left side of each line. Arrowheads indicate VLP binding on the epithelium, for which is characterized by brown staining. For the duodenum, magnified areas are indicated by dashed boxes (magnification x400). GII.17 variants are indicated above the panels.

Virus-like particles have previously been used to demonstrate that HBGAs are the natural ligands of HuNoVs using saliva binding assays and histological assays (Marionneau et al., 2002; Green et al., 2020). The role of the HBGA was later confirmed in enterocytes where HBGAs are highly expressed and HuNoVs replicate (Karandikar et al., 2016). Here, we intestinal tissue to study GII.17-HBGA interactions. For the GII.17 variants including the CS-E1 variant, we observed VLP attachment to the duodenal mucosa (Figure 7, panels A–C). However, binding intensity was lower for CS-E1 VLPs. In addition, binding was abolished when the duodenal tissues were pretreated with NaIO_4_, demonstrating the importance of glycan structures (data not shown). Inversely, no binding was observed for sections of the distal colon where HBGAs are also expressed, albeit in lower concentration (Figure 7, panels D–F; Ravn and Dabelsteen, 2000). Here, the first observations were concordant with saliva binding assays for the Kawasaki 308 and 323 variants. Inversely, for the CS-E1 variant, weak VLP detection in duodenal tissues was discordant with the absence of VLP binding in saliva binding assays. This observation suggests that saliva binding assays were somewhat limited for the analysis of poor HBGA binders like the GII.17 CS-E1 variant.

### HBGA Characterization on Duodenal Tissues

**Figure 8:**
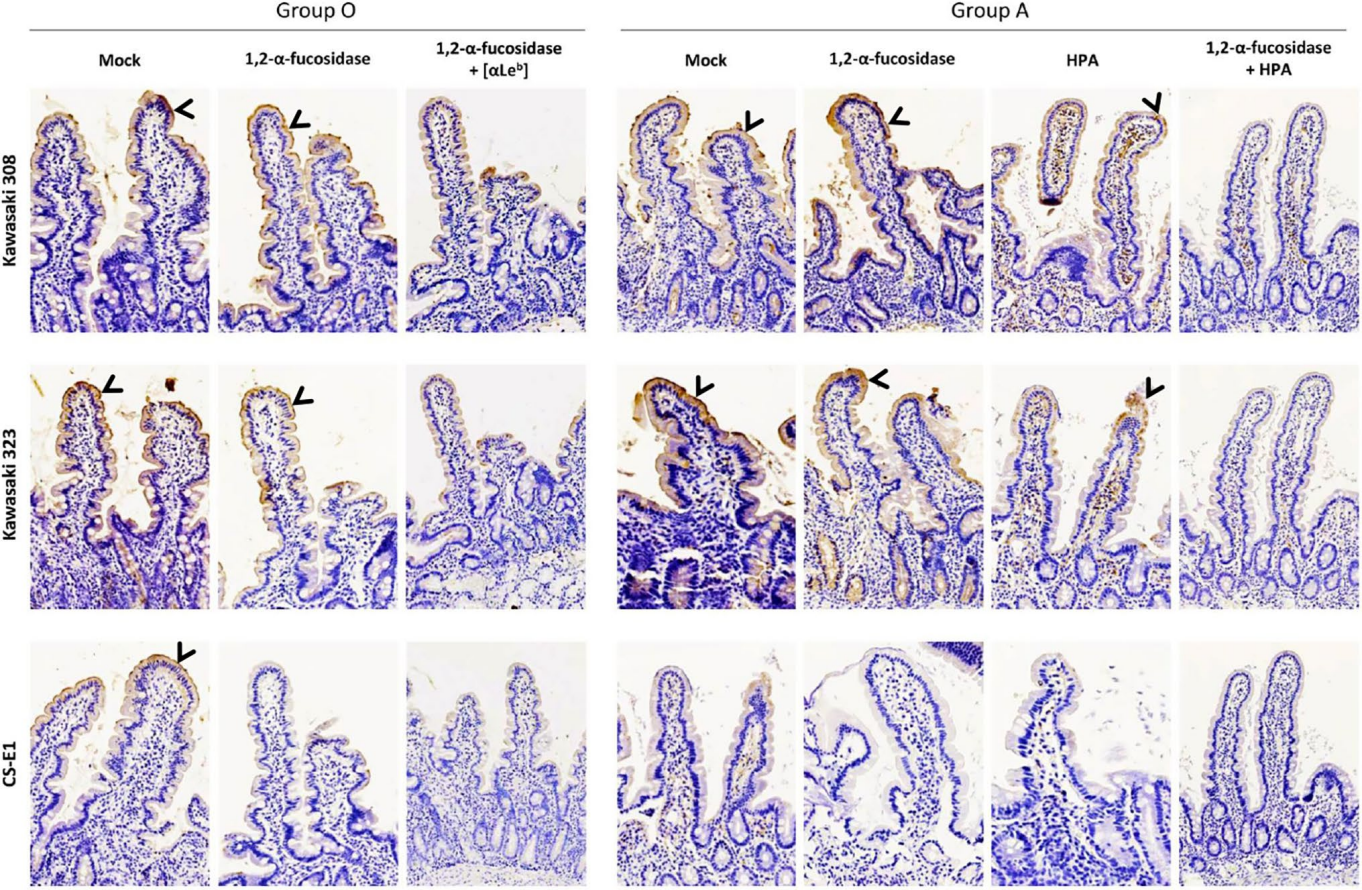
Histological analysis on duodenal sections of GII.17 recognized by HBGA. Duodenal tissue samples from group O and A individuals were used for VLP binding assays and competition experiments where sections were singly or concomitantly incubated with 1,2-α-L-fucosidase, Le^b^-specific mAb (anti-Le^b^) and HPA. Patient blood group and GII.17 variant are indicated above the panel series. For the competition experiments, fucosidase, mAb, and lectin are indicated on the left side of each panel series. Positive VLP detection is featured by brown staining and indicated by arrowheads on all images.

We used healthy secretor duodenal sections from group O and A donors to characterize GII.17 attachment to HBGA (Figure 8). For the group O individual, the duodenal mucosa strongly expressed the Le^b^ antigen but was negative for Le^a^ (Supplementary Figure 2), attesting to the secretor phenotype of the donor. The VLPs derived from the three variants specifically attached to the epithelium although the binding intensity for the CS-E1 variant was very faint and mainly located in the apical pole of villous cells. Active 1,2-α-L-fucosidase totally suppressed CS-E1 binding to the epithelium, suggesting that the α1,2-fucose moiety was involved in the recognition of this variant (Figure 8). Surprisingly, VLPs from Kawasaki 308 and 323 variants still recognized the fucosidase-treated epithelium from the group O secretor (Le^a^-Le^b^+). We then hypothesized that Le^b^ might be involved into the recognition of GII.17 HuNoVs. Incubation of the duodenal section with a Le^b^-specific antibody abrogated attachment for Kawasaki 308 and 323 variants. At this point, histological analysis confirmed that recent GII.17 variants (i.e., Kawasaki 308 and 323 variants) were more efficient binders since they showed an extended binding profile encompassing A, B, H, and Lewis antigens (i.e., Le^b^). Additionally, for CS-E1 binding assays, our data suggested that the use of histological sections was physiologically more relevant than saliva binding assays.

In the next set of experiments, we characterized GII.17 binding using healthy duodenal sections from a blood group A donor. VLP binding was observed for the Kawasaki 308 and 323 variants but not for the CS-E1 variant. It is worth noting that the absence of binding for the CS-E1 variant was later confirmed with duodenal tissues from three other blood group A individuals (data not shown). Kawasaki 308 and 323 VLP binding was further characterized. Kawasaki 308 and 323 VLP attachment to the mucosa was partially abrogated following incubation with HPA while fucosidase pretreatment alone did not hamper VLP binding. However, the incubation of HPA on fucosidase-treated tissues totally abolished VLP attachment, as previously shown with saliva samples (Figure 6B). This also confirmed VLP attachment to tissue sections through HBGA recognition.

## Discussion

Human norovirus is a major cause of gastroenteritis in all age groups worldwide. GII.4 has been by far the most predominant genotype for the last 30 years (Siebenga et al., 2007, 2009; van Beek et al., 2018; Cannon et al., 2021). The GII.17 genotype emerged in 2014–2015 and became predominant concomitantly with the GII.4 2012 variant, especially in Asia. It was then hypothesized that the predominant GII.4 genotype might be replaced by GII.17 genotypes (de Graaf et al., 2015). However, the switch between those genotypes did not occur, and GII.17 failed to become predominant. Here, we characterized the HBGA binding properties of three known variants of GII.17 HuNoV in comparison with those of the GII.4 2012 variant, which circulated at the same time. The main objective of the study was to determine why GII.17 suddenly emerged in many countries. The successful propagation of the GII.17 genotype occurred as the new Kawasaki 308 variant emerged. Genetic analysis showed that GII.17 isolates belonged to three distinct variants, which vary widely, as previously noticed for GII.4 variants. Capsid amino acid sequences were well conserved within each variant while the analysis of sequence alignment showed several amino acid deletions between variants, mainly located in the hypervariable region. Our study focused on the interactions between GII.17 and HBGAs since it was initially observed that the emergent GII.17 Kawasaki attached to HBGAs present in individuals of the secretor phenotype more or less irrespective of their ABO type, unlike earlier GII.17 strains (Chan et al., 2015; Zhang et al., 2015). Our data clearly show that GII.17 binding capacity to HBGA increased with time and the most recent Kawasaki 308 variant showed the strongest attachment to HBGA (Table 1). Altogether, our data highlight that the evolution of GII.17 HuNoVs was characterized by an increasing capacity to interact with HBGA.

**Table 1:** GII.17 interactions with HBGAs.

	HBGA (number of saliva samples)	GII.17 variant			GII.4 variant
		CS-E1	Kawasaki 323	Kawasaki 308	2012
Saliva binding assay (OD450)^a^	O (26)	<0.2	0.90 ± 0.23	2.39 ± 0.47	ND
	AB (6)	<0.2	1.26 ± 0.19	3.06 ± 0.16	ND
	A (16)	<0.2	1.21 ± 0.20 (1)^d^	2.93 ± 0.21 (1)^d^	ND
	B (20)	<0.2	0.91 ± 0.24 (1)^d^	2.45 ± 0.85	ND
	NS (33)	<0.2	<0.2	<0.2	ND
Surface plasmon resonance (RU)^b^	LNFP-I (H)	ND	ND	255	1,457
	A	ND	ND	−5	1
	B	ND	ND	−2	75
	Le^x^	ND	ND	−1	1
Immunohistological analysis^c^	O	+	+++	+++	ND
	A	−	+++	+++	ND
	AB	+	+++	+++	ND

a Optical density at 450 nm (OD450) below 0.2 (<0.2): no VLP binding. The OD shown here is the mean of the OD values above 0.2. SD, standard deviation and ND, not determined.

b The values are given in resonance unit (RU).

c For duodenal tissues, the intensity of the signal is graded based upon a positive control from a previous study (Tarris et al., 2021). +++, strong; ++, intermediate; +, low/focal; and −, negative.

d Number of negative sample for the binding assay is indicated in parentheses.

For this study, like others in the literature, we largely relied upon the use of synthetic VLPs and saliva samples to study HuNoV–host interaction. We acknowledge that VLPs cannot entirely mimic native HuNoV particle behavior, as exemplified by the absence of CS-E1 VLP binding to saliva. The data suggested that either the VLPs were not properly assembled or that CS-E1 used an alternative ligand to HBGA. Furthermore, a relative affinity experiment using BSA-conjugated carbohydrates gave incomplete information about HuNoV-HBGA interactions considering that only the H antigen was recognized by GII.17 VLPs. It is clear that SPR and ELISA using synthetic carbohydrates provide interesting information about HuNoV binding capacity. Nevertheless, they may not entirely reflect the carbohydrate presentation on human tissues. To verify this hypothesis, binding assays were then performed using histological sections. Here, binding of the three GII.17 variants all involved HBGA. Therefore, the data suggests that, similar to assays in synthetic carbohydrates, saliva assays might not entirely reflect the binding status and poor binders, such as CS-E1, might not possess the capacity to attach to HBGA from saliva. Our data are coherent with previous studies showing that saliva binding status might not entirely reflect binding capability at the intestinal level considering that HBGA conformation and concentration might vary between intestinal and saliva samples (Ruvoen-Clouet et al., 2014; Ayouni et al., 2015). In addition, the recent use of enteroids suggested that saliva-based assay using VLP might not entirely reflect physiological conditions, especially those encountered during acute gastroenteritis (Ettayebi et al., 2016). We also acknowledge that our histological data are very preliminary and the analysis of a larger number of histological tissues should be undertaken to clearly determine GII.17 binding profiles within the population. That being said, the use of histological sections might be considered as a good alternative for the study of HuNoV-HBGA interactions in the absence of HIEs, which use is expensive and technically challenging for many laboratories. Histological tissues might thus be a good alternative to HIEs for the analysis of weak binders. The absence of binding observed for duodenal tissues from blood group A individual using CS-E1 VLPs, in contrast with observed binding for Kawasaki 323 and most of all 308 variants, suggested that HBGA binding capacity increased with the evolution of GII.17. Similarly, quantitative analysis of the binding highlighted the higher binding capacity of the most recent Kawasaki 308 variant when compared to Kawasaki 323. Again, higher binding efficiency to HBGA accompanied the evolution of GII.17, as also described for post-2000 GII.4 variants (de Rougemont et al., 2011). Molecular analyses showed that the replacement of the Valine residue at position 444 in the CS-E1 variant by a Tyrosine residue at position 442 (Kawasaki 323) or 444 (Kawasaki 308) is involved into a better recognition of the α1,2 fucose and a subsequent increased affinity to HBGA (Singh et al., 2015; Koromyslova et al., 2017; Qian et al., 2019).

The GII.17 genotype had been described but was rarely involved in gastroenteritis outbreaks before the emergence of the most recent variants (Zheng et al., 2006; Iritani et al., 2010). Since 2015, epidemiological surveys reveal a co-circulation of two major genotypes (i.e., GII.4 and GII.17) rather than GII.4 being entirely replaced by GII.17 (van Beek et al., 2018). Later reports show that, after being detected in large numbers from 2014 through 2017, GII.17 disappeared in 2018, being replaced by GII.4 2012 variant. If sterilizing immunity is largely involved into the emergence/disappearance of a new strain (Donaldson et al., 2008), then why do GII.4 variants persist while other emerging genotypes circulate for a limited time? Immunity alone cannot explain the success of GII.4 genotype for the last four decades. Our data indicate that the evolution GII.17 involves an increasing affinity toward their natural ligands, HBGAs. Still, binding efficacy for GII.17 remains lower than that observed for the GII.4 2012 variant. Increased but still lower affinity toward HBGA might partly explain why GII.17 did not persist. In the past, we showed that GII.42006b variants might be considered a “super strain” based on their binding to HBGA ligands, and we hypothesized that higher affinity and an extended binding spectrum of the GII.4 genotype contributed to its success (de Rougemont et al., 2011).

Unlike recent GII.4 variants, the HBGA binding spectrum in GII.17 appears more restricted and of lower relative affinity, as observed using synthetic carbohydrates. In recent years, epidemiological surveys have shown that GII.17 was less predominant, correlating with the reoccurrence of GII.4 2012 variant associated with a new polymerase type (Lindesmith et al., 2018).

In summary, molecular surveys can be used to shed light on the emergence of non-GII.4 noroviruses. GII.17 is one example of a non-GII.4 HuNoV genotype that become predominant for a couple of years before disappearing. It could be hypothesized that a primary condition for replacing the predominant GII.4 strain is a higher (or at least equal) HBGA binding capacity. Today, the molecular survey of human noroviruses is as important as ever, seeing the emergence and reemergence of old strains with increased pathogenicity, as exemplified by the GII.17 variants assessed in this study. It has been shown that future predominant GII.4 variants circulated at low levels before emerging, and they were not necessarily the byproduct of the epochal evolution of the current predominant circulating strain (Ruis et al., 2020). We may hypothesize that the same goes for non-GII.4 emerging strains, making it difficult to choose the non-GII.4 genotypes that should be included in vaccine formulations, since there is no clear evidence of group antigens across HuNoV genotypes. It is widely accepted that the GII.4 genotype should be included in future HuNoV vaccines, and discussion is warranted relative to the addition of non-GII.4 genotypes.

## Data Availability Statement

The datasets presented in this study can be found in online repositories. The names of the repository/repositories and accession number(s) can be found at: https://www.ncbi.nlm.nih.gov/genbank/, KU587626.

## Ethics Statement

The studies involving human participants were reviewed and approved by French national ethics committee (CPP19002). The patients/participants provided their written informed consent to participate in this study.

## Author Contributions

ME, GT, AdR, and GB: conceptualization. ME, GT, and GB: methodology. ME, GT, NA-H, AR, and RC: investigation. SA, PD-F, ME, and GT: resources. JP, WB, FB, and GB: validation. GB: writing original draft and supervision. JP, GT, and GB: review and editing. AdR and GB: funding acquisition. GT, LM, ME, AdR, and GB: data curation. All authors contributed to the article and approved the submitted version.

## Funding

This study was partially funded by Santé Publique France, the National reference Center for Viral Gastroenteritis and the public hospital of Dijon. The authors acknowledge the support of biophysical and nanocharacterization facilities of the Clinical-Innovation Proteomic Platform (CLIPP, Besançon, France). At the time of the study, Siwar Ayouni was sponsored by a fellowship from Campus France.

## Conflict of Interest

The authors declare that the research was conducted in the absence of any commercial or financial relationships that could be construed as a potential conflict of interest.

## Publisher’s Note

All claims expressed in this article are solely those of the authors and do not necessarily represent those of their affiliated organizations, or those of the publisher, the editors and the reviewers. Any product that may be evaluated in this article, or claim that may be made by its manufacturer, is not guaranteed or endorsed by the publisher.

## References

[ref1] Ahmed S. M. Hall A. J. Robinson A. E. Verhoef L. Premkumar P. Parashar U. D.. (2014). Global prevalence of norovirus in cases of gastroenteritis: a systematic review and meta-analysis. Lancet Infect. Dis. 14, 725–730. doi: 10.1016/S1473-3099(14)70767-4, PMID: 24981041 PMC8006533

[ref2] Ayouni S. Estienney M. Sdiri-Loulizi K. Ambert-Balay K. De Rougemont A. Aho S.. (2015). Relationship between GII.3 norovirus infections and blood group antigens in young children in Tunisia. Clin. Microbiol. Infect. 21, 874.e871–878.e871. doi: 10.1016/j.cmi.2015.05.015 26003283

[ref3] Banyai K. Estes M. K. Martella V. Parashar U. D. (2018). Viral gastroenteritis. Lancet 392, 175–186. doi: 10.1016/S0140-6736(18)31128-0, PMID: 30025810 PMC8883799

[ref4] Belliot G. Lopman B. A. Ambert-Balay K. Pothier P. (2014). The burden of norovirus gastroenteritis: an important foodborne and healthcare-related infection. Clin. Microbiol. Infect. 20, 724–730. doi: 10.1111/1469-0691.12722, PMID: 24943671 PMC7962369

[ref5] Belliot G. Noel J. S. Li J.-F. Seto Y. Humphrey C. D. Ando T.. (2001). Characterization of capsid genes, expressed in the Baculovirus system, of three new genetically distinct strains of a Norwalk-Like viruses. J. Clin. Microbiol. 39, 4288–4295. doi: 10.1128/JCM.39.12.4288-4295.2001, PMID: 11724834 PMC88538

[ref6] Bull R. A. Eden J. S. Rawlinson W. D. White P. A. (2010). Rapid evolution of pandemic noroviruses of the GII.4 lineage. PLoS Pathog. 6:e1000831. doi: 10.1371/journal.ppat.1000831, PMID: 20360972 PMC2847951

[ref7] Cannon J. L. Bonifacio J. Bucardo F. Buesa J. Bruggink L. Chan M. C.. (2021). Global trends in Norovirus genotype distribution among children with acute gastroenteritis. Emerg. Infect. Dis. 27, 1438–1445. doi: 10.3201/eid2705.204756, PMID: 33900173 PMC8084493

[ref8] Chan M. C. Lee N. Hung T. N. Kwok K. Cheung K. Tin E. K.. (2015). Rapid emergence and predominance of a broadly recognizing and fast-evolving norovirus GII.17 variant in late 2014. Nat. Commun. 6:10061. doi: 10.1038/ncomms10061, PMID: 26625712 PMC4686777

[ref9] Chhabra P. De Graaf M. Parra G. I. Chan M. C. Green K. Martella V.. (2019). Updated classification of norovirus genogroups and genotypes. J. Gen. Virol. 100, 1393–1406. doi: 10.1099/jgv.0.001318, PMID: 31483239 PMC7011714

[ref10] de Graaf M. Van Beek J. Vennema H. Podkolzin A. T. Hewitt J. Bucardo F.. (2015). Emergence of a novel GII.17 norovirus - end of the GII.4 era? Eur. Secur. 20, 8–15. doi: 10.2807/1560-7917.es2015.20.26.21178 PMC5921880 26159308

[ref11] de Rougemont A. Ruvoen-Clouet N. Simon B. Estienney M. Elie-Caille C. Aho S.. (2011). Qualitative and quantitative analysis of the binding of GII.4 Norovirus variants onto human blood group antigens. J. Virol. 85, 4057–4070. doi: 10.1128/JVI.02077-10, PMID: 21345963 PMC3126233

[ref12] Donaldson E. F. Lindesmith L. C. Lobue A. D. Baric R. S. (2008). Norovirus pathogenesis: mechanisms of persistence and immune evasion in human populations. Immunol. Rev. 225, 190–211. doi: 10.1111/j.1600-065X.2008.00680.x, PMID: 18837783

[ref13] Ettayebi K. Crawford S. E. Murakami K. Broughman J. R. Karandikar U. Tenge V. R.. (2016). Replication of human noroviruses in stem cell-derived human enteroids. Science 353, 1387–1393. doi: 10.1126/science.aaf5211, PMID: 27562956 PMC5305121

[ref14] Fu J. Ai J. Jin M. Jiang C. Zhang J. Shi C.. (2015). Emergence of a new GII.17 norovirus variant in patients with acute gastroenteritis in Jiangsu, China, September 2014 to March 2015. Euro Surveill. 20:21157. doi: 10.2807/1560-7917.es2015.20.24.21157, PMID: 26111236

[ref15] Green K. Y. (2007). “Caliciviridae: the noroviruses,” in Fields Virology. eds. Knipe D. M. Howley P. M.. 5th *Edn.* (Philadelphia, PA: Lippincott, Williams & Wilkins), 949–980.

[ref16] Green K. Y. Kaufman S. S. Nagata B. M. Chaimongkol N. Kim D. Y. Levenson E. A.. (2020). Human norovirus targets enteroendocrine epithelial cells in the small intestine. Nat. Commun. 11:2759. doi: 10.1038/s41467-020-16491-3, PMID: 32488028 PMC7265440

[ref17] Havelaar A. H. Kirk M. D. Torgerson P. R. Gibb H. J. Hald T. Lake R. J.. (2015). World Health Organization global estimates and regional comparisons of the burden of foodborne disease in 2010. PLoS Med. 12:e1001923. doi: 10.1371/journal.pmed.1001923 26633896 PMC4668832

[ref18] Iritani N. Kaida A. Kubo H. Abe N. Goto K. Ogura H.. (2010). Molecular epidemiology of noroviruses detected in seasonal outbreaks of acute nonbacterial gastroenteritis in Osaka City, Japan, from 1996-1997 to 2008-2009. J. Med. Virol. 82, 2097–2105. doi: 10.1002/jmv.21915, PMID: 20981799

[ref19] Jin M. Zhou Y. K. Xie H. P. Fu J. G. He Y. Q. Zhang S.. (2016). Characterization of the new GII.17 norovirus variant that emerged recently as the predominant strain in China. J. Gen. Virol. 97, 2620–2632. doi: 10.1099/jgv.0.000582, PMID: 27543110 PMC5756485

[ref20] Kambhampati A. Payne D. C. Costantini V. Lopman B. A. (2016). Host genetic susceptibility to enteric viruses: a systematic review and meta analysis. Clin. Infect. Dis. 62, 11–18. doi: 10.1093/cid/civ873, PMID: 26508510 PMC4679673

[ref21] Karandikar U. C. Crawford S. E. Ajami N. J. Murakami K. Kou B. Ettayebi K.. (2016). Detection of human norovirus in intestinal biopsies from immunocompromised transplant patients. J. Gen. Virol. 97, 2291–2300. doi: 10.1099/jgv.0.000545, PMID: 27412790 PMC5756488

[ref22] Katayama T. Sakuma A. Kimura T. Makimura Y. Hiratake J. Sakata K.. (2004). Molecular cloning and characterization of bifidobacterium bifidum 1,2-alpha-L-fucosidase (AfcA), a novel inverting glycosidase (glycoside hydrolase family 95). J. Bacteriol. 186, 4885–4893. doi: 10.1128/JB.186.15.4885-4893.2004, PMID: 15262925 PMC451662

[ref23] Koromyslova A. Tripathi S. Morozov V. Schroten H. Hansman G. S. (2017). Human norovirus inhibition by a human milk oligosaccharide. Virology 508, 81–89. doi: 10.1016/j.virol.2017.04.032, PMID: 28505592

[ref24] Kumar S. Stecher G. Li M. Knyaz C. Tamura K. (2018). MEGA X: molecular evolutionary genetics analysis across computing platforms. Mol. Biol. Evol. 35, 1547–1549. doi: 10.1093/molbev/msy096, PMID: 29722887 PMC5967553

[ref25] Lindesmith L. C. Brewer-Jensen P. D. Mallory M. L. Debbink K. Swann E. W. Vinje J.. (2018). Antigenic characterization of a novel recombinant GII.P16-GII.4 Sydney Norovirus strain With minor sequence variation leading to antibody escape. J. Infect. Dis. 217, 1145–1152. doi: 10.1093/infdis/jix651, PMID: 29281104 PMC5939617

[ref26] Lindesmith L. Moe C. Marionneau S. Ruvoen N. Jiang X. Lindblad L.. (2003). Human susceptibility and resistance to Norwalk virus infection. Nat. Med. 9, 548–553. doi: 10.1038/nm860, PMID: 12692541

[ref27] Loureiro Tonini M. A. Pires Goncalves Barreira D. M. Bueno De Freitas Santolin L. Bondi Volpini L. P. Gagliardi Leite J. P. Le Moullac-Vaidye B.. (2020). FUT2, secretor status and FUT3 polymorphisms of children with acute diarrhea infected with rotavirus and norovirus in Brazil. Viruses 12:1084. doi: 10.3390/v12101084 PMC7600990 32992989

[ref28] Lozano R. Naghavi M. Foreman K. Lim S. Shibuya K. Aboyans V.. (2012). Global and regional mortality from 235 causes of death for 20 age groups in 1990 and 2010: a systematic analysis for the global burden of disease study 2010. Lancet 380, 2095–2128. doi: 10.1016/S0140-6736(12)61728-0, PMID: 23245604 PMC10790329

[ref29] Lu J. Sun L. Fang L. Yang F. Mo Y. Lao J.. (2015). Gastroenteritis outbreaks caused by Norovirus GII.17, Guangdong Province, China, 2014-2015. Emerg. Infect. Dis. 21, 1240–1242. doi: 10.3201/eid2107.150226, PMID: 26080037 PMC4480401

[ref30] Marionneau S. Ruvoën N. Le Moullac-Vaidye B. Clement M. Cailleau-Thomas A. Ruiz-Palacois G.. (2002). Norwalk virus binds to histo-blood group antigens present on gastroduodenal epithelial cells of secretor individuals. Gastroenterology 122, 1967–1977. doi: 10.1053/gast.2002.33661, PMID: 12055602 PMC7172544

[ref31] Matsui T. Hamako J. Ozeki Y. Titani K. (2001). Comparative study of blood group-recognizing lectins toward ABO blood group antigens on neoglycoproteins, glycoproteins and complex-type oligosaccharides. Biochim. Biophys. Acta 1525, 50–57. doi: 10.1016/S0304-4165(00)00170-7, PMID: 11342253

[ref32] Matsumoto I. Osawa T. (1969). Purification and characterization of an anti-H(O) phytohemagglutinin of Ulex europeus. Biochim. Biophys. Acta 194, 180–189. doi: 10.1016/0005-2795(69)90193-7, PMID: 5353123

[ref33] Matsushima Y. Ishikawa M. Shimizu T. Komane A. Kasuo S. Shinohara M.. (2015). Genetic analyses of GII.17 norovirus strains in diarrheal disease outbreaks from December 2014 to March 2015 in Japan reveal a novel polymerase sequence and amino acid substitutions in the capsid region. Euro Surveill. 20:21173. doi: 10.2807/1560-7917.es2015.20.26.21173 26159307

[ref34] Mori K. Motomura K. Somura Y. Kimoto K. Akiba T. Sadamasu K. (2017). Comparison of genetic characteristics in the evolution of Norovirus GII.4 and GII.17. J. Med. Virol. 89, 1480–1484. doi: 10.1002/jmv.24791, PMID: 28198556

[ref35] Prokop O. Uhlenbruck G. Kohler W. (1968). A new source of antibody-like substances having anti-blood group specificity. A discussion on the specificity of helix agglutinins. Vox Sang. 14, 321–333. doi: 10.1111/j.1423-0410.1968.tb01722.x, PMID: 5660844

[ref36] Qian Y. Song M. Jiang X. Xia M. Meller J. Tan M.. (2019). Structural adaptations of Norovirus GII.17/13/21 lineage through two distinct evolutionary paths. J. Virol. 93, e01655–e01718. doi: 10.1128/JVI.01655-18, PMID: 30333166 PMC6288326

[ref37] Rackoff L. A. Bok K. Green K. Y. Kapikian A. Z. (2013). Epidemiology and evolution of rotaviruses and noroviruses from an archival WHO global study in children (1976-79) with implications for vaccine design. PLoS One 8:e59394. doi: 10.1371/journal.pone.0059394, PMID: 23536875 PMC3607611

[ref38] Ravn V. Dabelsteen E. (2000). Tissue distribution of histo-blood group antigens. APMIS 108, 1–28. doi: 10.1034/j.1600-0463.2000.d01-1.x, PMID: 10698081

[ref39] Ruis C. Lindesmith L. C. Mallory M. L. Brewer-Jensen P. D. Bryant J. M. Costantini V.. (2020). Preadaptation of pandemic GII.4 noroviruses in unsampled virus reservoirs years before emergence. Virus Evol. 6:veaa067. doi: 10.1093/ve/veaa067, PMID: 33381305 PMC7751145

[ref40] Ruvoen-Clouet N. Magalhaes A. Marcos-Silva L. Breiman A. Figueiredo C. David L.. (2014). Increase in genogroup II.4 norovirus host spectrum by CagA-positive helicobacter pylori infection. J. Infect. Dis. 210, 183–191. doi: 10.1093/infdis/jiu054, PMID: 24459192

[ref41] Scallan E. Hoekstra R. M. Angulo F. J. Tauxe R. V. Widdowson M. A. Roy S. L.. (2011). Foodborne illness acquired in the United States: major pathogens. Emerg. Infect. Dis. 17, 7–15. doi: 10.3201/eid1701.P11101, PMID: 21192848 PMC3375761

[ref42] Shoemaker G. K. Van Duijn E. Crawford S. E. Uetrecht C. Baclayon M. Roos W. H.. (2010). Norwalk virus assembly and stability monitored by mass spectrometry. Mol. Cell. Proteomics 9, 1742–1751. doi: 10.1074/mcp.M900620-MCP200, PMID: 20418222 PMC2938053

[ref43] Siebenga J. J. Vennema H. Renckens B. De Bruin E. Van Der Veer B. Siezen R. J.. (2007). Epochal evolution of GGII.4 norovirus capsid proteins from 1995 to 2006. J. Virol. 81, 9932–9941. doi: 10.1128/JVI.00674-07, PMID: 17609280 PMC2045401

[ref44] Siebenga J. J. Vennema H. Zheng D.-P. Vinjé J. Lee B. E. Pang X.-L.. (2009). Norovirus illness is a global problem: emergence and spread of norovirus GII.4 variants, 2001–2007. J. Infect. Dis. 200, 802–812. doi: 10.1086/605127, PMID: 19627248

[ref45] Singh B. K. Koromyslova A. Hefele L. Gurth C. Hansman G. S. (2015). Structural evolution of the emerging 2014-2015 GII.17 Noroviruses. J. Virol. 90, 2710–2715. doi: 10.1128/JVI.03119-15, PMID: 26699640 PMC4810701

[ref46] Tarris G. Belliot G. Callier P. Huet F. Martin L. De Rougemont A. (2019). Pathology of rotavirus-driven multiple organ failure in a 16-month-old boy. Pediatr. Infect. Dis. J. 38, e326–e328. doi: 10.1097/INF.0000000000002472, PMID: 31634298

[ref47] Tarris G. De Rougemont A. Estienney M. Charkaoui M. Mouillot T. Bonnotte B.. (2021). Specific norovirus interaction with lewis x and lewis a on human intestinal inflammatory mucosa during refractory inflammatory bowel disease. mSphere 6:e01185-20. doi: 10.1128/mSphere.01185-20 33441404 PMC7845605

[ref48] Tenge V. R. Hu L. Prasad B. V. V. Larson G. Atmar R. L. Estes M. K.. (2021). Glycan recognition in human Norovirus infections. Viruses 13:2066. doi: 10.3390/v13102066, PMID: 34696500 PMC8537403

[ref49] van Beek J. De Graaf M. Al-Hello H. Allen D. J. Ambert-Balay K. Botteldoom N.. (2018). Molecular surveillance of norovirus, 2005-16: an epidemiological analysis of data collected from the NoroNet network. Lancet Infect. Dis. 18, 545–553. doi: 10.1016/S1473-3099(18)30059-8, PMID: 29396001

[ref50] Vinje J. Estes M. K. Esteves P. Green K. Y. Katayama K. Knowles N. J.. (2019). ICTV virus taxonomy profile: Caliciviridae. J. Gen. Virol. 100, 1469–1470. doi: 10.1099/jgv.0.001332, PMID: 31573467 PMC7011698

[ref51] Wang L. P. Zhou S. X. Wang X. Lu Q. B. Shi L. S. Ren X.. (2021). Etiological, epidemiological, and clinical features of acute diarrhea in China. Nat. Commun. 12:2464. doi: 10.1038/s41467-021-22551-z 33927201 PMC8085116

[ref52] Zhang X. F. Huang Q. Long Y. Jiang X. Zhang T. Tan M.. (2015). An outbreak caused by GII.17 norovirus with a wide spectrum of HBGA-associated susceptibility. Sci. Rep. 5:17687. doi: 10.1038/srep17687, PMID: 26639056 PMC4671059

[ref53] Zheng D. P. Ando T. Fankhauser R. L. Beard R. S. Glass R. I. Monroe S. S. (2006). Norovirus classification and proposed strain nomenclature. Virology 346, 312–323. doi: 10.1016/j.virol.2005.11.015, PMID: 16343580

