# Supplementary figures and images for: Epidemiological Impact of GII.17 Human Noroviruses Associated With Attachment to Enterocytes
Source: Front Microbiol. 2022 Apr 27;13:858245. doi: 10.3389/fmicb.2022.858245 (PMC9094630; doi:10.3389/fmicb.2022.858245)

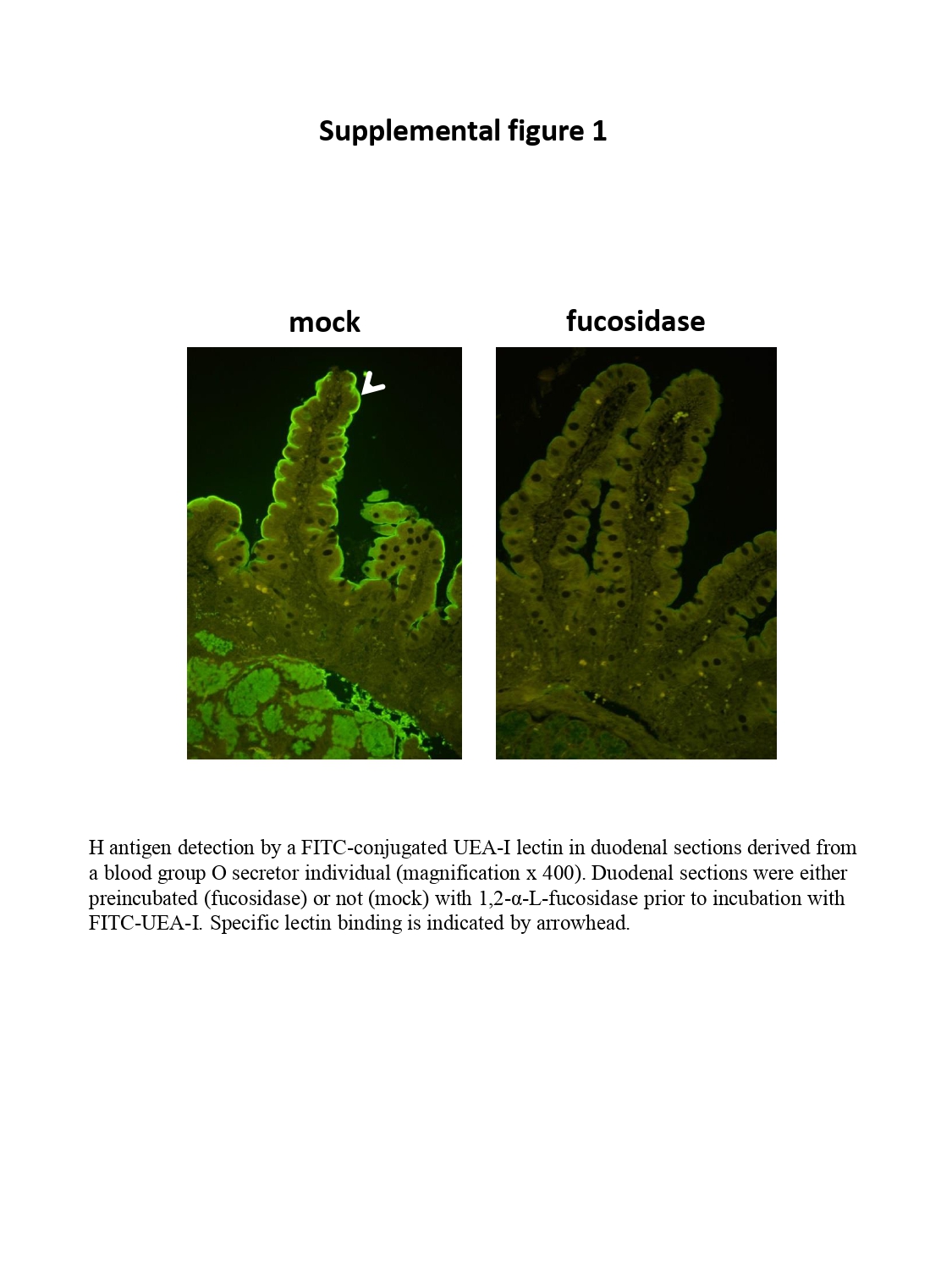

Supplement: Supplementary file 1 [file Image_1.JPEG]

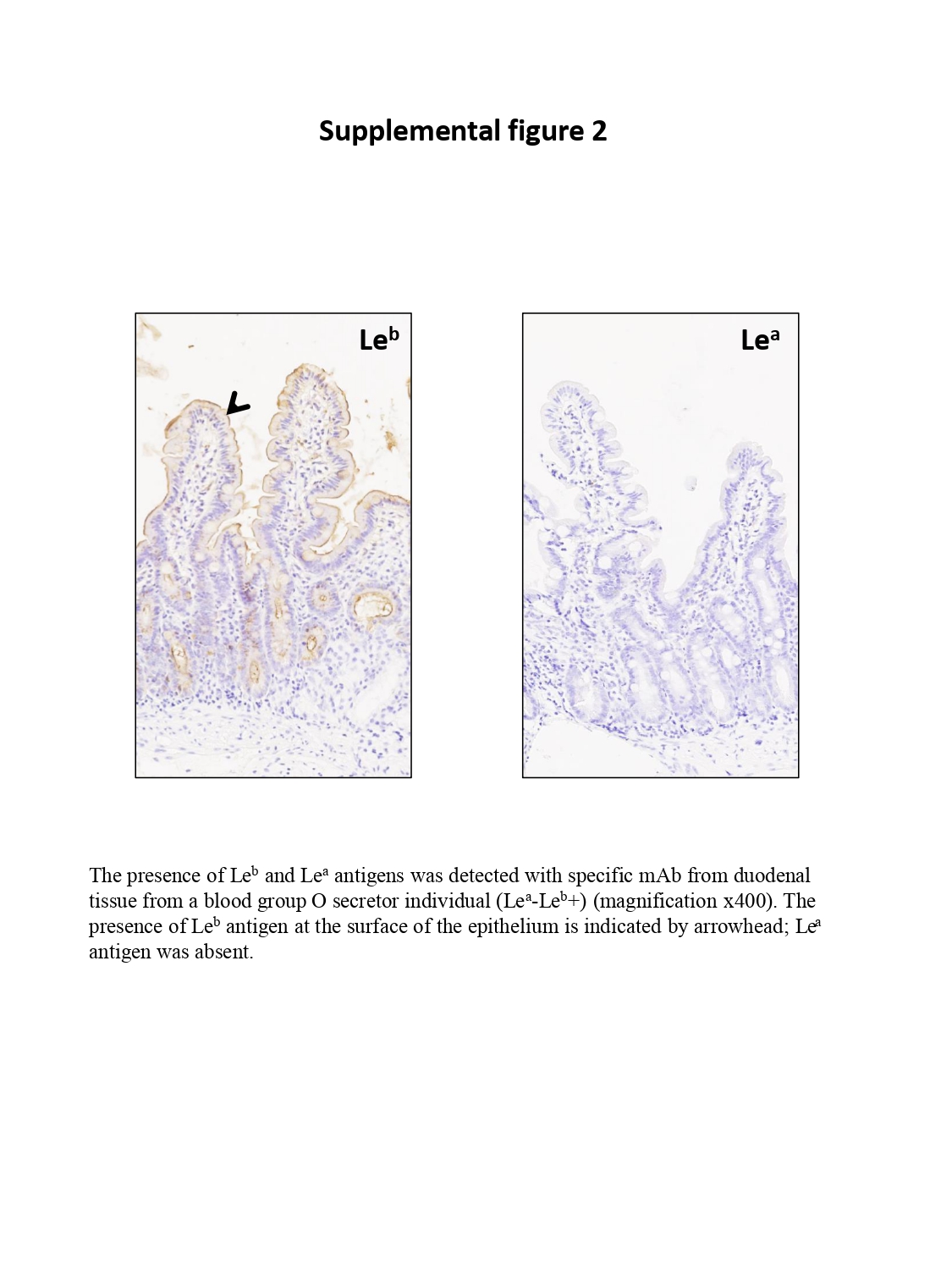

Supplement: Supplementary file 2 [file Image_2.jpg]
